# Supplementary material for: Photogating-assisted tunneling boosts the responsivity and speed of heterogeneous WSe2/Ta2NiSe5 photodetectors
Source: Nat Commun. 2024 Jan 2;15:141. doi: 10.1038/s41467-023-44482-7 (PMC10762006; doi:10.1038/s41467-023-44482-7)
Supplement: Supplementary file 1 — Supplementary Information [file 41467_2023_44482_MOESM1_ESM.pdf]

# Supplementary Information

# Photogating-Assisted Tunneling Boosts the Responsivity and Speed of Heterogeneous $WSe_2/Ta_2NiSe_5$ Photodetectors

Mingxiu Liu,[1,2, //] Jingxuan Wei,[3,4 //] Liujian Qi,[1,2, //] Junru An,[1,2] Xingsi Liu,[3] Yahui Li,[1,2] Zhiming Shi,[1,2] Dabing Li,[1,2,]* Kostya S. Novoselov,[5] Cheng-Wei Qiu,[3,]* Shaojuan Li[1,2,]*

[1]State Key Laboratory of Luminescence and Applications, Changchun Institute of Optics, Fine Mechanics and Physics, Chinese Academy of Sciences, Changchun, Jilin 130033, P. R. China

[2]University of Chinese Academy of Sciences (UCAS), Beijing 100049, P. R. China

[3]Department of Electrical and Computer Engineering, National University of Singapore, Singapore 117583, Singapore

[4]School of Optoelectronic Science and Engineering, University of Electronic Science and Technology of China, Chengdu 611731, China

[5]Institute for Functional Intelligent Materials, National University of Singapore, Singapore 117544, Singapore

*Corresponding author. E-mail: (D. L.) lidb@ciomp.ac.cn; (C.-W. Q.) chengwei.qiu@nus.edu.sg and (S. L.) lishaojuan@ciomp.ac.cn;

// These authors contributed equally.

The Supplementary Information contains 23 display items.

The structural characterization by angle-resolved polarized Raman spectroscopy, SEM and EDS, and notes for the anisotropic structural determination of the $Ta_2NiSe_5$ crystals are provided. Details of the Raman mapping and PL mapping images, the derivation process and detailed analysis of the electrical properties of $Ta_2NiSe_5$ and $WSe_2$, the gate tunable photoresponse of $WSe_2/Ta_2NiSe_5$ heterostructure, numerical simulations of the band energy distribution, details of the device photoresponse at different wavelengths, characterization of $WSe_2/Ta_2NiSe_5$ heterostructure with different thicknesses, polarized photoresponse of the device are also contained.

## Supplementary Note 1. Anisotropic structural characterization of $Ta_2NiSe_5$ crystals.

The structures of $Ta_2NiSe_5$ are schematically illustrated in **Supplementary Fig. 1** and **Supplementary Fig. 2a**, in which the Ta double chain with octahedral coordination and Ni single chain with tetrahedral coordination run along the *a* axis. Furthermore, two [$TaSe_6$] octahedral single chains interconnected with $NiSe_4$ single chains are periodically assembled along the *c* axis direction.

Prior to the device fabrication, we first determined the in-plane crystal axis (i.e., *a* and *c* axis) of $Ta_2NiSe_5$, which is beneficial to the further research of anisotropic optoelectrical properties of $Ta_2NiSe_5$. The representative optical microscope images of the $Ta_2NiSe_5$ flakes are shown in **Supplementary Fig. 2b**. Due to their anisotropic crystal structure,[1, 2] the $Ta_2NiSe_5$ crystals typically appear to be a rectangular-like shape after exfoliation during our experiments. The angle-resolved polarized Raman spectroscopy can be utilized as a non-contact and non-destructive method for directly identifying the orientation of $Ta_2NiSe_5$ crystals. Therefore, the identification of the *a*-axis and *c*-axis of our samples can be identified. As shown in **Supplementary Fig. 2c**, the long-axis of the crystal is defined as the y direction in the experimental coordinate, the x direction is perpendicular to the y-axis, the angle $\theta$, i.e., the incident angle between the polarization directions of the incident light and x-axis of the crystal, was varied from 0 to 360°. The different Raman modes intensities change periodically with the variation of rotation angle under the parallel-polarized configurations, indicating a strong in-plane vibration anisotropy (**Supplementary Fig. 2d**). The characteristic Raman peaks of $Ta_2NiSe_5$ were identified from the spectrum. The Raman active modes of 96.6, 121.1, 147, 176, 190, and 214 $cm^{-1}$ are assigned to $A_g^1$ to $A_g^6$, respectively, which is consistent with previous reports.[3, 4] As shown in **Supplementary Fig. 2e**, the peak intensity can be written as: $I(A_g^1) = ae^{i\varphi_{ad}}cos^2(\theta) + 2dsin\theta cos\theta + be^{i\varphi_{bd}}sin^2(\theta)$, the fact that the intensity of Raman peaks changed periodically with $\theta$ from 0 to 180° is noteworthy, and the same changing trend was confirmed in multiple samples. The $A_g^1$ mode is minimized when the direction of laser polarization is parallel to the y direction (i.e., the long axis of the flake), which corresponds to *a*-axis of the crystal, according to previous reports.[1]

To verify the difference in conductivity between the two orthogonal directions, we also fabricated a multi-electrode device based on the $Ta_2NiSe_5$ crystal, as shown in **Supplementary Fig. 2f**. The crystallographic direction of the flake is confirmed by angle-resolved polarized Raman spectroscopy. We found that the current along the *a*-axis direction (≈ $2\times10^{-3}$ A at $V_{ds}$=1

V) is significantly higher than that along the *c*-axis direction ($\approx 5\times10^{-4}$ A at $V_{ds}$=1 V). According to above measurements, a convenient way to determine the crystal orientation is by inspecting the crystal shape, and the crystal is generally elongated along the *a* axis of the crystal.

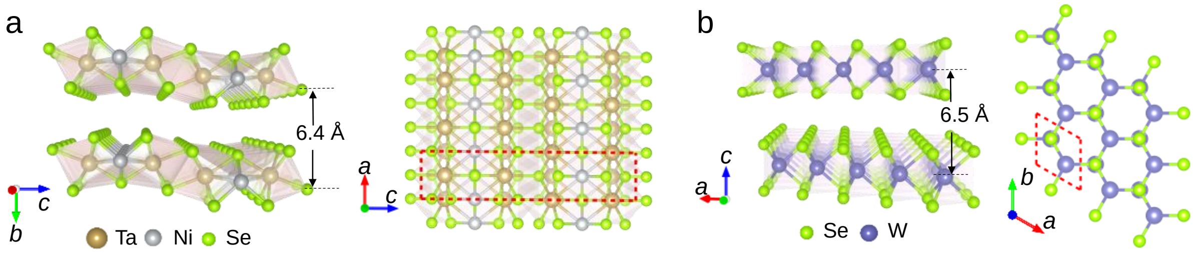


**Supplementary Fig. 1** Schematic of the crystal structures of **a**, $Ta_2NiSe_5$, and **b**, $WSe_2$.

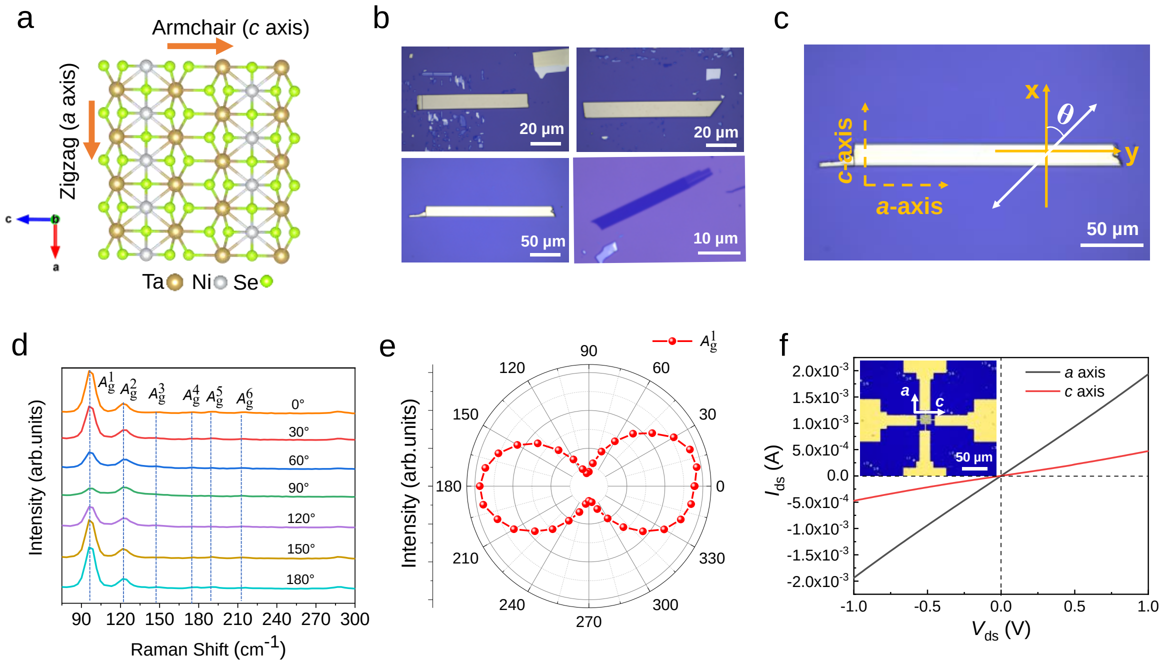


**Supplementary Fig. 2 Anisotropic structural characterization of $Ta_2NiSe_5$ crystals**. **a**, Schematic of the anisotropic structure of $Ta_2NiSe_5$. **b-c**, Optical microscope images of the exfoliated $Ta_2NiSe_5$ flakes. The white arrow represents the direction of polarization of light, the angle between the white arrow and the x-axis is defined as the polarization angle $\theta$. **d**, The angle-resolved polarized Raman spectra of $Ta_2NiSe_5$. **e**, The peak intensity ($A_g^1$ mode) as a function of polarization angle. **f**, The anisotropic electrical transport property of $Ta_2NiSe_5$. Inset: Top-view optical image of the fabricated multi-electrode $Ta_2NiSe_5$ device.

## Supplementary Note 2. Optical characterization of the $WSe_2/Ta_2NiSe_5$ heterostructure.

The coupling effect and charge transfer at $WSe_2/Ta_2NiSe_5$ interface can be verified via optical characterization. **Supplementary Fig. 3a** shows the non-polarized Raman spectra collected from isolated $WSe_2$, $Ta_2NiSe_5$, and the overlapped heterojunction region, respectively. The characteristic peaks of both $WSe_2$ and $Ta_2NiSe_5$ were observed in the overlapped heterostructure region. The corresponding Raman mapping images were measured at the characteristic Raman peaks of $Ta_2NiSe_5$ (121 $cm^{-1}$) and $WSe_2$ (260 $cm^{-1}$), respectively, as shown in **Supplementary Fig. 3b, c**. The Raman mapping images exhibit a good homogeneity of each component, indicating high quality of the heterostructure after the exfoliation and target-transfer processes. We also collected the PL spectra from different areas of the heterostructure with a laser illumination of 532 nm (**Supplementary Fig. 3d**). Isolated $Ta_2NiSe_5$ shows no obvious PL signal in the measured wavelength range due to its narrow optical bandgap.[3, 5] Two obvious peaks at ~780 nm (corresponding to ≈1.6 eV) and 860 nm (≈1.42 eV) are observed for $WSe_2$. These two PL peaks are related to the *A* excitons (*A*) and indirect transitions (*I*), which is in accordance with the PL spectra of multilayer $WSe_2$.[6, 7] Notably, the PL intensity at the heterojunction is much weaker than that of isolated $WSe_2$. The PL mapping image in **Supplementary Fig. 3e** further confirms the quenching effect of PL at the heterojunction area, indicating the efficient separation and transition of photogenerated electrons and holes.[8-10] In addition, **Supplementary Fig. 3f** shows the UV-vis–NIR absorption spectra of $Ta_2NiSe_5$, $WSe_2$ and $WSe_2/Ta_2NiSe_5$, respectively. The peak at ~760 nm of $WSe_2$ in the absorption spectrum is attributed to its strong excitonic absorption.[11] In contrast, multilayer $Ta_2NiSe_5$ has an obvious broad spectral absorption because of its narrow bandgap.

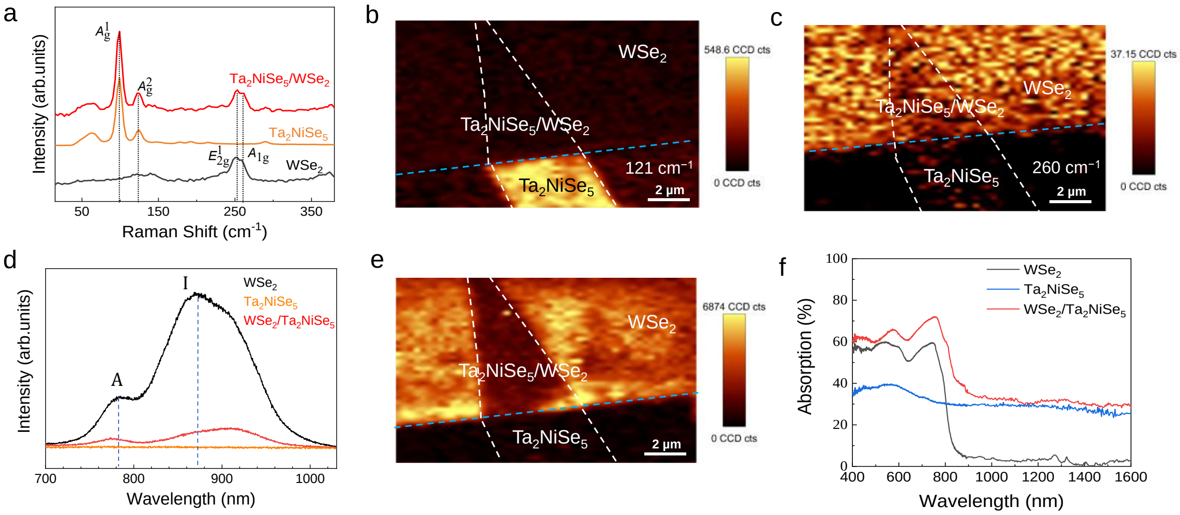


**Supplementary Fig. 3 Optical characterization of the $WSe_2/Ta_2NiSe_5$ heterojunction. a**, The non-polarized Raman spectra collected from isolated $WSe_2$, $Ta_2NiSe_5$, and the overlapped heterojunction region. **b-c**, The corresponding Raman mapping images measured at the characteristic Raman peaks of (b) $Ta_2NiSe_5$ (121 $cm^{-1}$) and (c) $WSe_2$ (260 $cm^{-1}$). The blue and white dashed lines represent the regions of $WSe_2$ and $Ta_2NiSe_5$, respectively. **d**, PL spectra of isolated $WSe_2$, $Ta_2NiSe_5$, and the $WSe_2/Ta_2NiSe_5$ heterojunction. The dashed line corresponds to the position of the exciton absorption peak of $WSe_2$. **e**, The corresponding PL mapping image of the $WSe_2/Ta_2NiSe_5$ heterojunction. **f**, UV-vis–NIR absorption spectra of $Ta_2NiSe_5$, $WSe_2$ and $WSe_2/Ta_2NiSe_5$.

## Supplementary Note 3. Thickness characterization and elemental distribution of the $WSe_2$/ $Ta_2NiSe_5$ heterojunctions

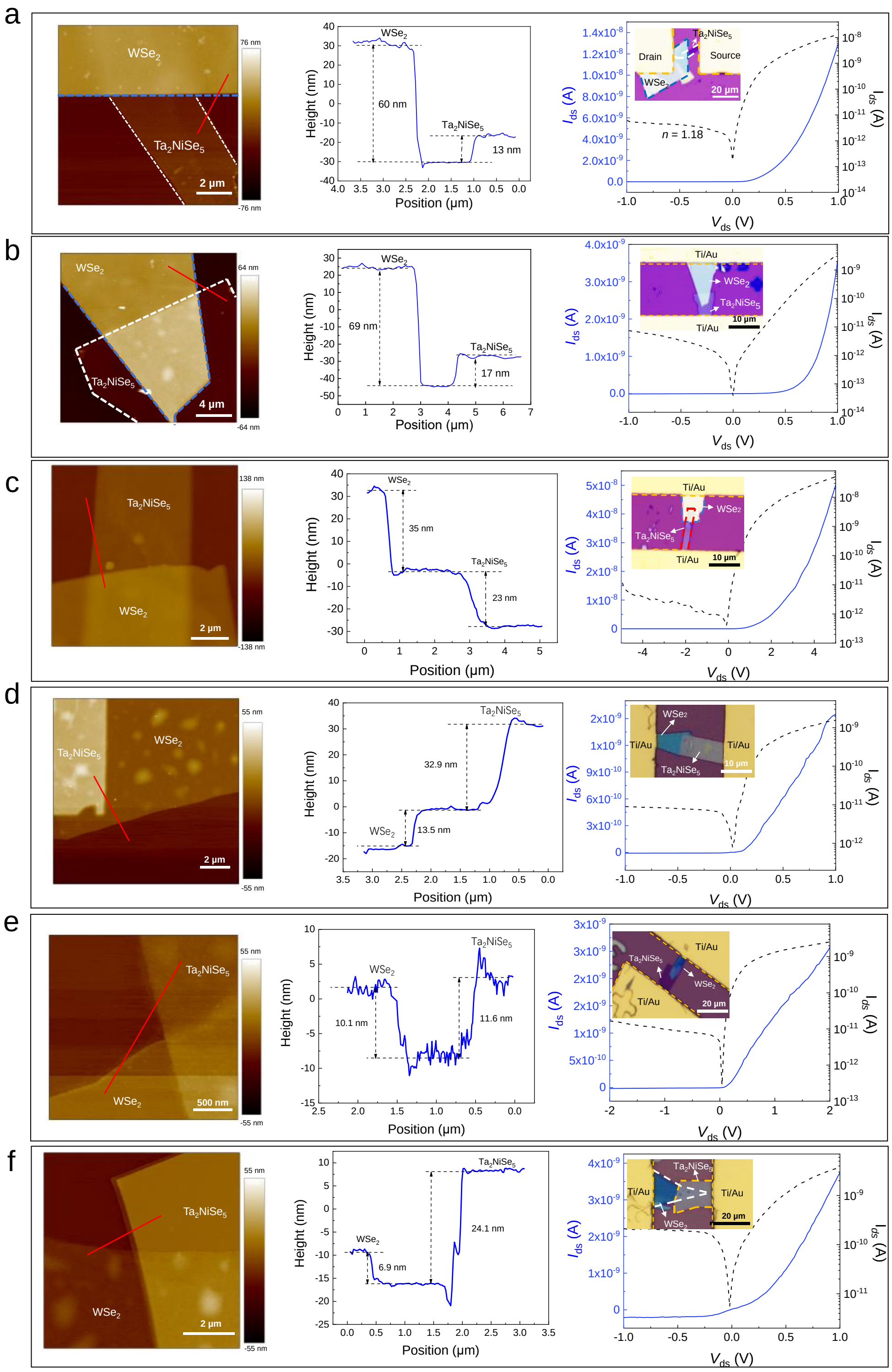


**Supplementary Fig. 4 Characterization of the $WSe_2$/$Ta_2NiSe_5$ heterojunction of different thicknesses.**

**Supplementary Fig. 5a** shows the scanning electron microscope (SEM) image of a typical device. The energy-dispersive spectroscopy (EDS) spectrum was measured at the $Ta_2NiSe_5$ and $WSe_2$ region, respectively (**Supplementary Fig. 5b, c**). The results give strong signals of Ta, Ni, Se, and W, Se, respectively, and basically agree with the stoichiometric ratio with slight selenium vacancy.

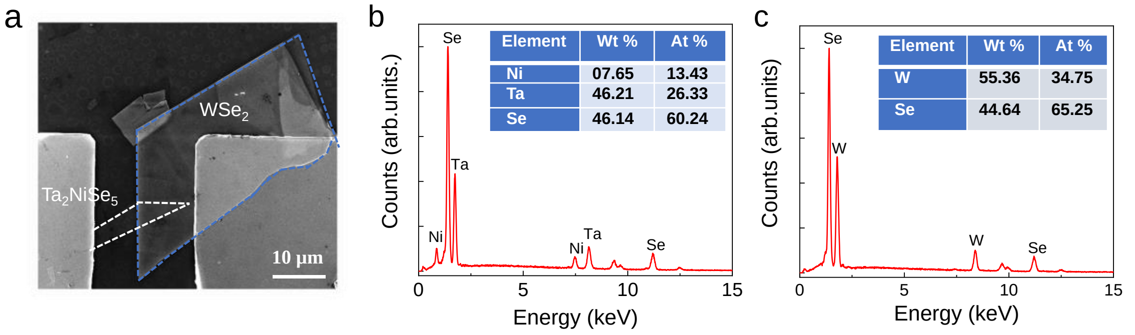


**Supplementary Fig. 5 Characterization of the $WSe_2/Ta_2NiSe_5$ heterojunction. a**, SEM image taken at the heterostructure region. The blue and white dashed lines represent the regions of $WSe_2$ and $Ta_2NiSe_5$, respectively. **b-c**, The corresponding EDS results of $Ta_2NiSe_5$ and $WSe_2$, respectively.

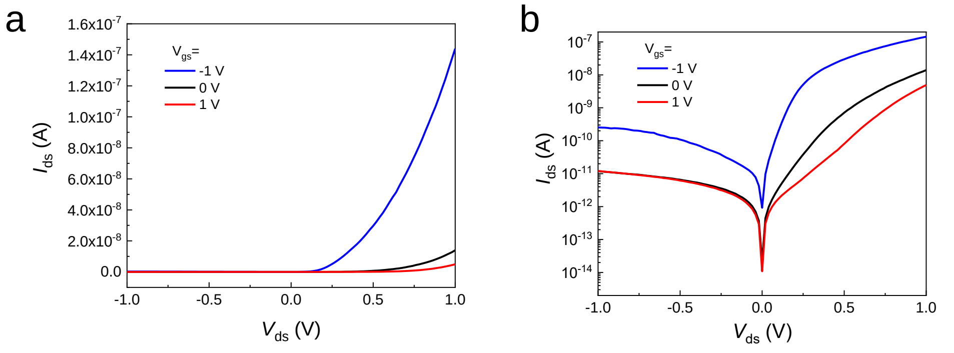


**Supplementary Fig. 6 Output characteristics ($I_{ds}$-$V_{ds}$) of the $WSe_2/Ta_2NiSe_5$ heterostructure device. a,** linear scale and **b,** log-scale of the curve.

## Supplementary Note 4. Electrical characterization of $Ta_2NiSe_5$ and $WSe_2$ field effect transistors.

To investigate the electrical polarity of $Ta_2NiSe_5$ and $WSe_2$ respectively, we fabricated $Ta_2NiSe_5$ and $WSe_2$ field effect transistors (FETs) on Si/$SiO_2$ substrate and then performed the electrical measurements at room temperature. **Supplementary Fig. 7a, e** shows the optical images of the back-gated $Ta_2NiSe_5$ and $WSe_2$ with Ti/Au source/drain electrodes, respectively. The $WSe_2$ and $Ta_2NiSe_5$ flakes were cleaved by the mechanical exfoliation and transferred on the Si/$SiO_2$ (300 nm) substrate. The morphology and thickness of the flakes were characterized using AFM, and it can be observed that the surface of the sample is very flat (**Supplementary Fig. 7b, f**). As is shown in **Supplementary Fig. 7c, g**, the thickness of the $Ta_2NiSe_5$ and $WSe_2$ flakes were determined to be ~19 nm and 86 nm, respectively. **Supplementary Fig. 7d** shows the transfer curve of the $Ta_2NiSe_5$ FET with a linear plot of the source-drain current ($I_{ds}$) versus back gate voltage ($V_{gs}$) when sweeping from −5 to +5 V at a fixed source–drain voltage ($V_{ds}$ = 1 V). The transfer curve suggests the weakly *n*-type semiconducting property of $Ta_2NiSe_5$. Furthermore, we found that $Ta_2NiSe_5$ has a near ohmic contact with the metal electrode as shown in the inset of **Supplementary Fig. 7d**. On the other hand, the transfer curve of $WSe_2$ transistors at $V_{ds}$ = 1 V displays a typical hole-dominated *p*-type semiconducting property (**Supplementary Fig. 7h**). The output curves at different back-gate voltages are shown in the inset of **Supplementary Fig. 7h**. The output current gradually decreases as the gate voltage changes from negative to positive, further proving that holes are the majority carriers in the material. Moreover, the $I_{ds}$–$V_{ds}$ curve under small gate bias shows that a Schottky contact was formed between $WSe_2$ and Ti/Au electrode in our experiments.

The carrier mobility ($\mu_{FE}$) of $Ta_2NiSe_5$ and $WSe_2$ were further extracted from the transfer curves, using a relation given as

$$\mu_{\mathrm{FE}} = \frac{L}{W}\left(\frac{\mathrm{d}I_{\mathrm{ds}}}{\mathrm{d}V_{\mathrm{gs}}}\right)\frac{1}{C_{\mathrm{bg}}V_{\mathrm{ds}}} \tag{1}$$

where *L* and *W* represent the channel length and width of the device, $\left(\frac{\mathrm{d}I_{ds}}{\mathrm{d}V_{\mathrm{gs}}}\right)$ gives the slope of the transfer curves, $C_{bg}$ (~ 115 aF/μm$^2$) is the gate capacitance. Thus, the $\mu_{FE}$ of $Ta_2NiSe_5$ and $WSe_2$ were estimated to be 12.43 and 4.64 cm$^2$/V·s, respectively.

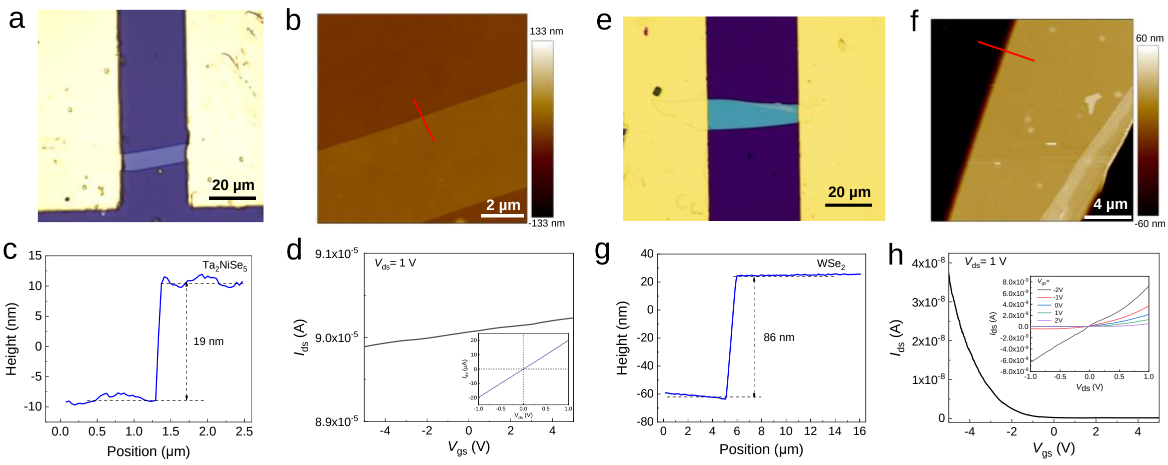


**Supplementary Fig 7 Electrical characterization of $Ta_2NiSe_5$ and $WSe_2$ FETs. a**, Optical image of the back-gated $Ta_2NiSe_5$ FET. **b**, AFM topography of the $Ta_2NiSe_5$ FET. **c**, Thickness measurement taken along the red solid line in b. **d**, Transfer curve ($I_{ds}$-$V_{gs}$) of the $Ta_2NiSe_5$ FET. Inset: output curve ($I_{ds}$-$V_{ds}$) of the $Ta_2NiSe_5$ FET. **e**, Optical image of the back-gated $WSe_2$ FET. **f**, AFM topography of the $WSe_2$ FET. **g**, Thickness measurement taken along the red solid line in f. **h**, Transfer curve ($I_{ds}$-$V_{gs}$) of the $WSe_2$ FET. Inset: output curve ($I_{ds}$-$V_{ds}$) of the $WSe_2$ FET.

## Supplementary Note 5. Energy band distribution of $WSe_2$/$Ta_2NiSe_5$ heterostructures.

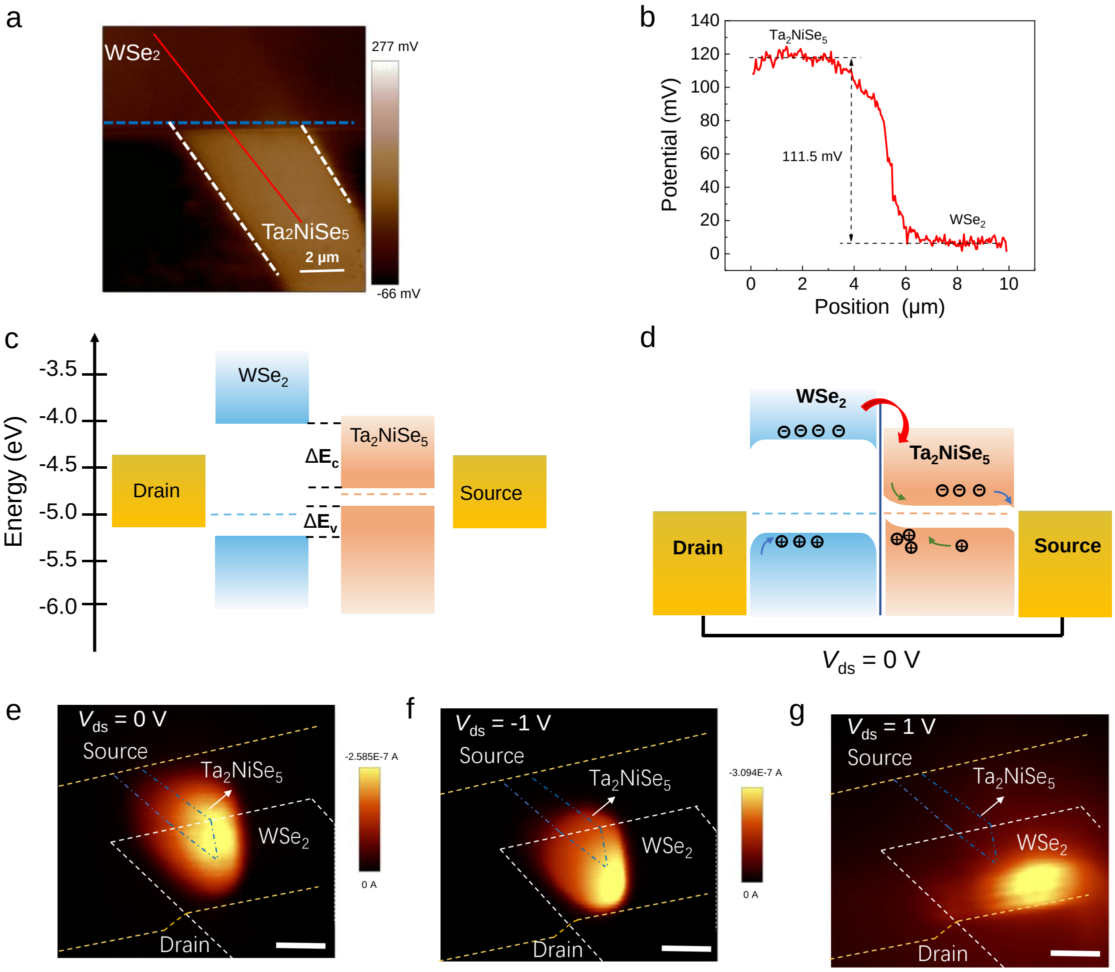


**Supplementary Fig. 8 Band diagram of the $WSe_2$/$Ta_2NiSe_5$ heterostructure device and corresponding photocurrent mapping images. a**, Kelvin probe force microscopy (KPFM) image that reveals the surface potential difference of the $WSe_2$/$Ta_2NiSe_5$ heterojunction. **b**, Surface potential difference taken along the red line in a. **c-d,** Band diagrams of the heterostructure device (c) before and (d) after contact. The arrow represents the direction of carrier transport. **e-g,** The spatially resolved photocurrent mapping images at (e) $V_{ds}$ = 0 V, (f) $V_{ds}$ = -1 V, and (g) $V_{ds}$ = 1 V. Scale bar: 6 μm. The illumination wavelength is 633 nm. The white, blue and yellow dashed lines represent the regions of $WSe_2$, $Ta_2NiSe_5$ and metal electrodes, respectively.

## Supplementary Note 6. Simulation on energy band distribution of few-layer $WSe_2/Ta_2NiSe_5$ heterostructures.

The band alignments of few-layer $WSe_2/Ta_2NiSe_5$ heterostructures were investigated by using density functional theory (DFT) methods. The Perdew-Burke-Ernzerhof (PBE) functional was adapted to describe electronic exchange-correlation interaction since its great reliability to transition metal dichalcogenides (TMDCs) including $MoS_2$, $PdSe_2$, $Pd_2Se_3$, InSe and so on.[12-14] The projected band structures of 1-6 layer $WSe_2/Ta_2NiSe_5$ heterostructures are shown in **Supplementary Fig. 9**. The conduction band minimum (CBM) and valence band maximum (VBM) of $Ta_2NiSe_5$ embed in the bandgap of $WSe_2$ despite of the thickness of $WSe_2$, indicating the type-I alignment, which perfectly agree with our experimental observations. Furthermore, the convergence of band structures to different thickness of $WSe_2$ implies the good predictability for thicker heterostructures.

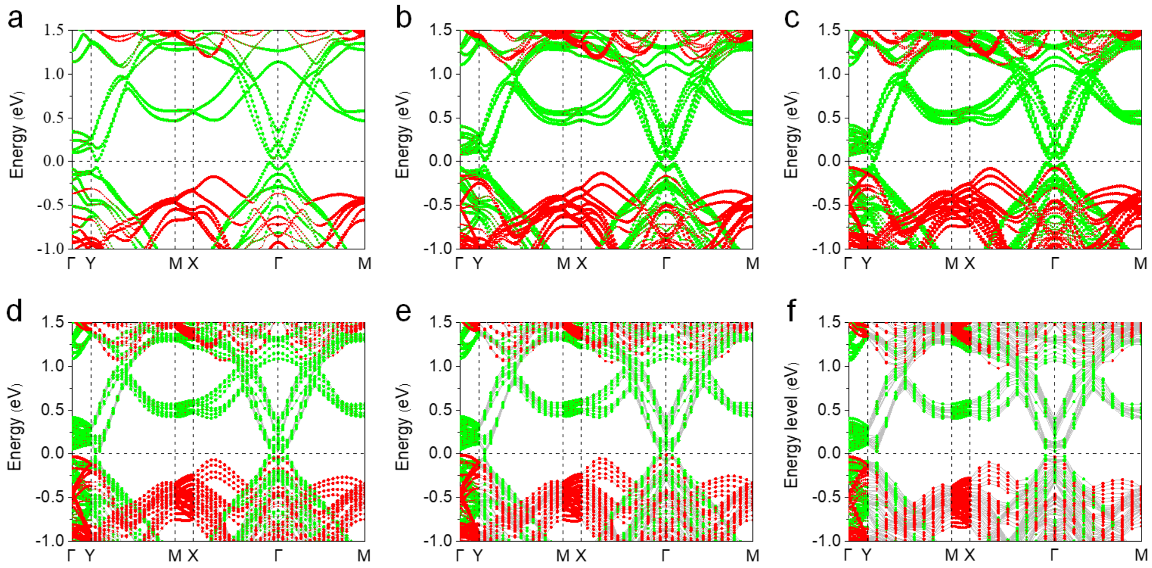


**Supplementary Fig. 9 The projected band structures of few-layer $WSe_2/Ta_2NiSe_5$ heterostructures. a-f** represents the heterostructures from monolayer 1L $Ta_2NiSe_5$/1L $WSe_2$ to six-layer 6L $Ta_2NiSe_5$/6L $WSe_2$. The green and red colors indicate the contributions of $Ta_2NiSe_5$ and $WSe_2$, respectively. The fermi level is set to 0 eV.

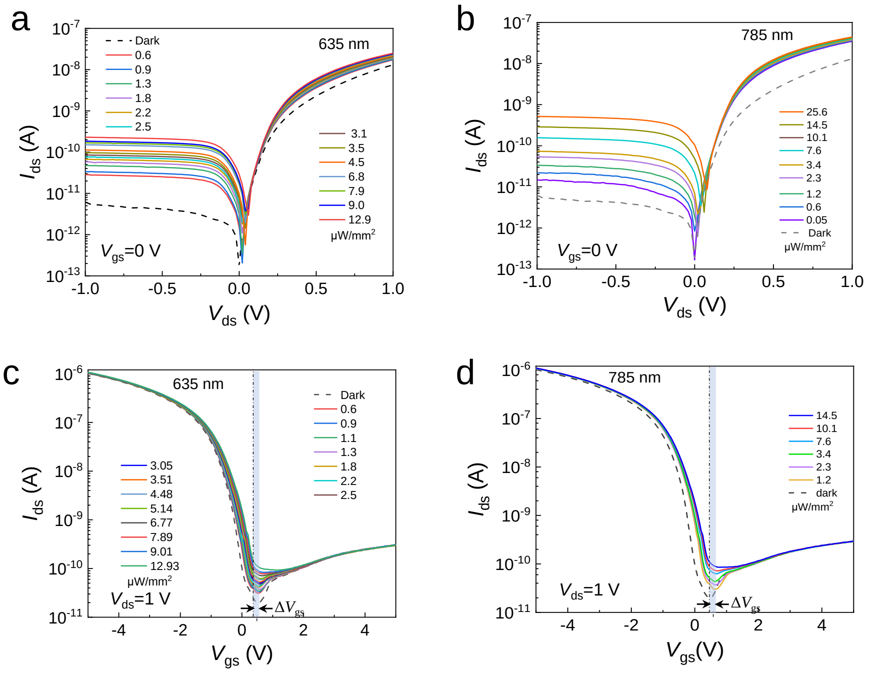


**Supplementary Fig. 10 Photoresponse of the $WSe_2/Ta_2NiSe_5$ heterostructure. a-b**, Output curves ($I_{ds}$-$V_{ds}$) in dark and under illumination with different power densities. The incident light is at 635 and 785 nm wavelength, respectively. $V_{gs}$ = 0 V. **c-d**, The corresponding transfer curves ($I_{ds}$-$V_{gs}$) of the device at $V_{ds}$= 1 V. $\Delta V_g$ and the shaded area represent the range of the change of the charge neutrality point.

## Supplementary Note 7. Photocarrier tunneling at positive bias voltage under light illumination.

The Schottky barrier in a Schottky junction essentially determines the junction's behavior by governing electron/hole transport. Depending on the height and width of the barrier, charge transport through the barrier may occur through thermionic emission (TE) or tunnelling, thereby we analyzed the device characteristics by using these two models in the following.

TE model is widely used to explain the electron transport mechanism of Schottky junction and to extract the Schottky barrier height.[15, 16] According to TE theory, density of current through the barrier is determined as

$$J = A^{*}T^{2}exp\left(-\frac{q\varphi_{B0}}{KT}\right)\left[exp\left(\frac{qV}{KT}\right) - 1\right] \quad (1)$$

where $A^{*}$ is the known Richardson constant in the previous study, which is 27.6 A $cm^{-2}$ $K^{-2}$ for the $WSe_2$.[17, 18] $T$ is temperature, $k$ is the Boltzmann constant, $q$ is the electronic charge, $V$ is the bias voltage applied. The saturation current is described as

$$J_0 = A^{*}T^{2}exp\left(-\frac{q\varphi_{B0}}{KT}\right) \quad (2)$$

where $\varphi_{B0}$ is the barrier height at zero bias. Experimentally, the saturation current can be estimated from the intersection of a linear approximation of ln *J(V)* and the Y-axis. So, $\varphi_{B0}$ is determined by equation

$$\varphi_{B0} = \frac{KT}{q}\ln\left(\frac{A^{*}T}{J_0}\right) \quad (3)$$

In real structures an *I-V* dependence deviates from the ideal theory, and an ideality factor $n$ is introduced:

$$J = J_0 exp\left(\frac{qV}{nKT}\right)\left[1 - exp\left(-\frac{qV}{KT}\right)\right] \quad (4)$$

According to the above formula, it is found that the barrier height ($q\varphi_{B0}$) and ideal factor $n$ of Schottky junction can be extracted from the intercept and slope of the ln *J*(*V)* curve in semi-logarithmic coordinates.[19]

$$q\varphi_{B0} = KT[A^{*}T - intercept] \quad (5)$$

$$n = \frac{q}{KT}\frac{dV}{d(\ln J)} \quad (6)$$

According to formulas (5) and (6), the calculated values of $n$ and $q\varphi_{B0}$ are 11.2 and 0.618 eV for the device, respectively (**Supplementary Fig. 11a**). However, it is found that the ideal factor $n$ is far greater than 1, indicating that there was an inaccuracy between the TE model and the experimental results.

Thereby, tunneling may dominate the transport of our device. It is noted that the $I_{ds}$-$V_{ds}$ curve at positive bias voltages can be well modeled by a tunneling barrier with the Simmons approximation (**Fig. 2f** and **Supplementary Fig. 11b, c**). The dominant tunneling occurs with direct tunneling (DT) at low bias voltage and Fowler–Nordheim tunneling (FNT) at high voltage. The DT and FNT can be expressed by[20-23]

$$I_{DT} \propto V exp\left(-\frac{4\pi d\sqrt{2m^*\varphi}}{h}\right) \quad (7)$$

$$I_{FNT} = V^2 exp\left(-\frac{8\pi d\sqrt{2m^*\varphi^3}}{3heV}\right) \quad (8)$$

Where $d$, $m$*, $\phi$, $h$ is the tunneling thickness, effective electron mass, tunneling barrier, and the Plank constant, respectively. The fitting plot of ln ($I/V^2$) versus $1/V$ shows linear dependence with a negative slope for the FNT under larger $V_{ds}$, and rises exponentially for the DT under small $V_{ds}$ under light illumination, as demonstrated in the figures. Similar phenomenon is also observed for other devices (**Supplementary Fig. 11d-f**, the device 3 as described in **Supplementary Note 9**). We also extract the corresponding parameters of device 3, and the calculated values of $n$ and $q\varphi_{B0}$ are 21.4 and 0.564 eV for the device respectively (**Supplementary Fig. 11d**). We found a similar phenomenon as **Supplementary Fig. 11a**, where the n value is far greater than 1. It is further verified that our device is not dominant by TE transport. The tunneling-mediated transport in the device 3 is also confirmed with a temperature-dependent measurements in **Supplementary Fig. 11e-f**. The plot of ln ($I/V^2$) versus $1/V$ displays small variations as the temperature decreases from 300 K to 128 K, with the linear region of curves exhibiting nearly the same slope. The above observations indicate that the tunneling-dominated transport of charge carriers under positive biases.

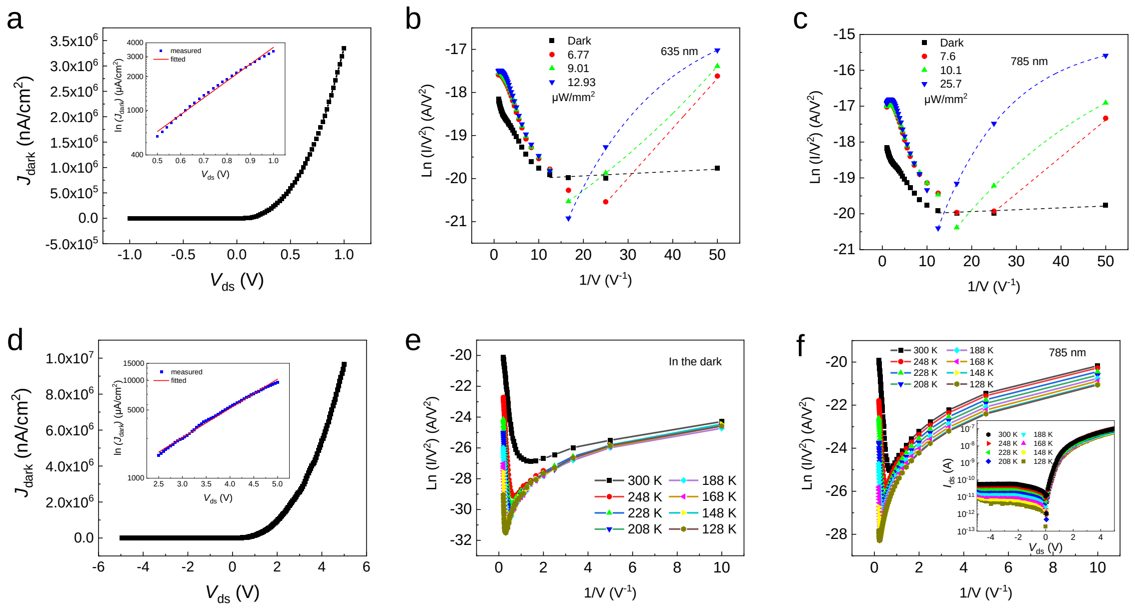

**Supplementary Fig. 11 Photocarrier tunneling at positive bias voltage. a,** $J_{dark}$-$V_{ds}$ curve of the $WSe_2/Ta_2NiSe_5$ heterostructure device in dark derived from Fig. 1c of the main text. Inset: ln $J(V)$-$V_{ds}$ plot of the device. The red line represents a linear fitting between current density and bias voltage in the positive bias range. **b**, Fowler–Nordheim plots of the device at $V_{ds}$ = 1 V in the dark and under light illumination of 635 nm. The dashed line represents the fits to the experimental data. **c,** Fowler–Nordheim plots of the device at $V_{ds}$ = 1 V in the dark and under light illumination of 785 nm. **d**, $J_{dark}$-$V_{ds}$ curve of a second device in dark. Inset: ln $J(V)$-$V_{ds}$ plot of the device. **e,** Temperature-dependent transport characteristics of the second device in the dark. **f,** Temperature-dependent transport characteristics of the second device under light illumination. Incident light power: 0.47 μW/mm$^2$. The incident wavelength is 785 nm.

## Supplementary Note 8. Photocarrier lifetime and transit time at different bias voltages.

Photodetection gain of a photodetector can be estimated from the device responsivity, following the below equation

$$G = \frac{h\upsilon}{e\eta} R \tag{1}$$

where $e$ is the electronic charge, $h$ is Planck's constant, and $v$ is the frequency of the incident photons, $\eta$ ~ 43% is the percentage of light absorption under the irradiation of 785 nm laser extracted from the absorption spectrum of $WSe_2$. The device responsivity $R = I_{ph}/(P{\cdot}S)$, where $I_{ph}$ is the photocurrent ($I_{ph} = I_{light} – I_{dark}$), $P$ is the power density and $S$ is the effective device area subjected to illumination. According to above **Supplementary Fig. 10**, the gain of the device can be calculated.

The carrier lifetimes were evaluated from the transient response of the device under pulsed light illumination. The decay curves of photocurrents when the incident light is turned off can be fitted by[24, 25]

$$I = I_0 + A\exp\left(-\frac{t}{\tau_{d1}}\right) + B\exp\left(-\frac{t}{\tau_{d2}}\right) \tag{2}$$

here $I_0$ denotes the saturation photocurrent, $A$ and $B$ are two constants, $\tau_{d1}$ and $\tau_{d2}$ are the fast and slow time constants for the decaying photocurrents, respectively. Based on equation (2), the values of $\tau_{d1}$ and $\tau_{d2}$ under light illumination at different bias conditions were obtained, respectively (**Supplementary Fig. 12**). The relatively fast process ($\tau_{d1}$) is attributed to the recombination of the carriers that occurs when the light is turned off, while the slow process ($\tau_{d2}$) is caused by detrapping of the carriers at defects/traps, which is corresponding to the carrier lifetime, $\tau$.

Then, the value of carrier transit time $t_L$ can be extracted according to $t_L = \tau / G$. The carrier transit time is 61 μs and 3.57 ns at -1 V and +1 V bias voltage under 785 nm illumination. It can be clearly seen that the carrier transit time of the device under positive bias is about four orders of magnitude faster than that under negative bias. Similar results are observed under incidence of other wavelengths .

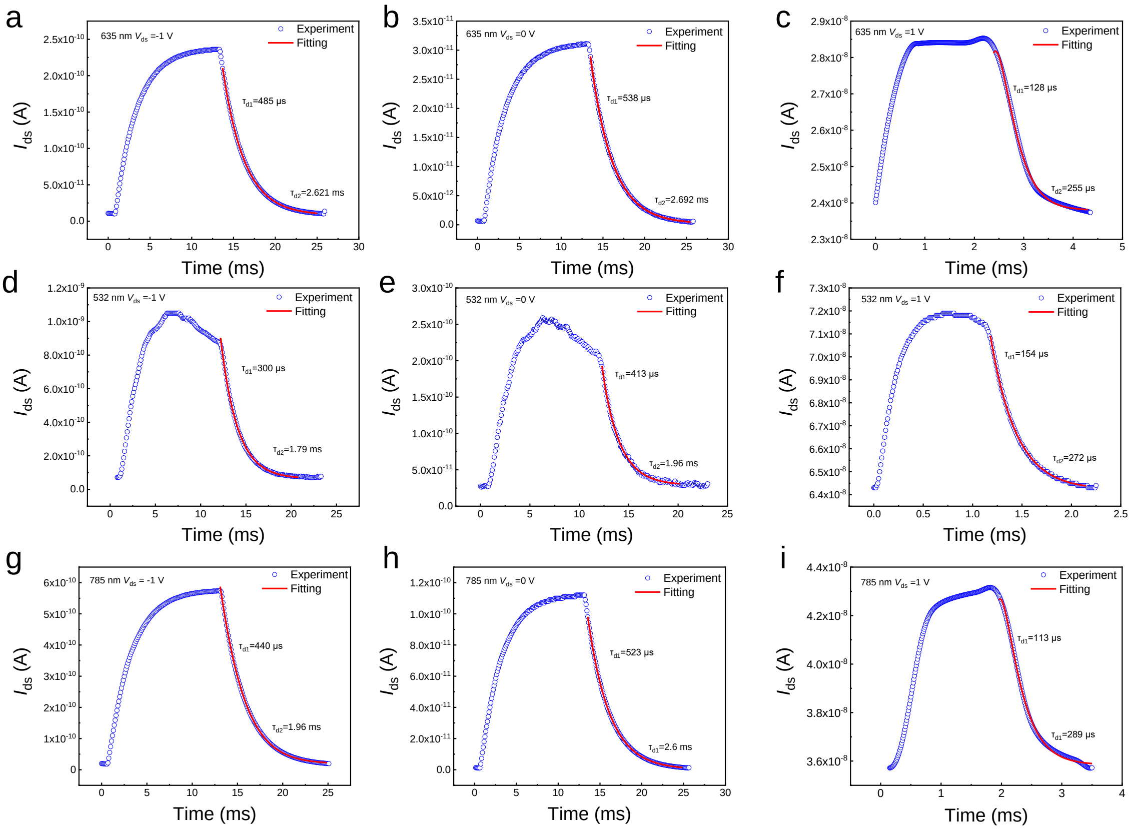


**Supplementary Fig. 12 The extracted carrier lifetime under light illumination at different bias conditions.** **a**, 635 nm, $V_{ds}$ = -1 V; **b**, 635 nm, $V_{ds}$ = 0 V; **c**, 635 nm, $V_{ds}$ = 1 V; **d**, 532 nm, $V_{ds}$ = -1 V; **e**, 532 nm, $V_{ds}$ = 0 V; **f**, 532 nm, $V_{ds}$ = 1 V; **g**, 785 nm, $V_{ds}$ = -1 V; **h**, 785 nm, $V_{ds}$ = 0 V; **i**, 785 nm, $V_{ds}$ = 1 V.

## **Supplementary Note 9. Photoresponse of $WSe_2/Ta_2NiSe_5$ heterostructures with different thicknesses.**

We fabricated heterostructure devices with different thicknesses of $Ta_2NiSe_5$ and $WSe_2$, and characterized the device optoelectronic properties, as demonstrated in **Supplementary Fig. 13-15**. First, we study the optoelectronic properties of thicker materials (corresponding to devices 1, 2 and 3), as shown in **Supplementary Table 1**, the thickness of the $WSe_2/Ta_2NiSe_5$ of device 1, 2 and 3 is 60 nm/13 nm, 69 nm/17 nm, 35 nm/23 nm respectively. Under light irradiation, all three devices exhibit obvious photoresponse. It is obvious that the responsivity of the device increases by about several orders of magnitude at the positive bias, in comparison to that under negative bias as shown in **Supplementary Fig. 13a, d, g**. For example, **Supplementary Fig. 13g** shows the responsivity of the device 1 at the positive bias (>$10^3$ A/W at 0.05 μW/mm$^2$), which is about three orders of magnitude larger than the values under negative bias (1.32 at 0.05 μW/mm$^2$). The dependence of photocurrent $I_{ph}$ on incident laser intensity $P$ was depicted in **Supplementary Fig. 13b, e, h**. It can be observed that the photocurrents of all devices have different power dependence under positive and negative bias. The $I_{ph}$ is proportional to $P^{\alpha}$ with a fitting value $\alpha$ = 0.8 at $V_{ds}$= -1 V (**Supplementary Fig. 13h)**. On the contrary, under 1 V bias voltage, the $\alpha$ is 0.2, which is significantly smaller than the optimal value ($\alpha$ = 1), indicating a large amount of traps or recombination centers exist in the device.[26-28] The time-resolved photoresponse of the device under pulsed light illumination were measured at different bias modes in **Supplementary Fig. 13c, f, i**. We found that the response time at positive bias is more than 10 times faster compared with the case under negative bias in **Supplementary Fig. 13i**.

To further verify the effect of material thickness on the device performance, we also fabricated heterojunction devices with thinner $WSe_2$, which are devices 4, 5 and 6, respectively. The thickness of $WSe_2$ in the device is 13.5,10.1 and 6.9 nm, respectively. **Supplementary Fig. 14a-i** show several devices characteristics under different $V_{ds}$, including important parameters such as responsivity, specific detectivity, and response time, which were assessed by measuring the photocurrent ($I_{ph}$) under 785 nm illuminated light with different powers. Compared with thicker devices, the responsivity of thin devices does not decrease significantly, and the maximum responsivity of thin devices is obviously greater than $10^3$ A/W in **Supplementary Fig. 14a, d, g**. The important thing is that the responsivity of positive bias voltage is several orders of magnitude higher than that of negative bias voltage. It shows the same phenomenon as the previously studied thick devices. For the response speed of several devices, we can clearly see that under the action of the tunneling mechanism, the response speed of the device

under the positive bias voltage is obviously higher than that of the negative bias voltage. For instance, Device 6 shows a rise time of 23.6 μs under positive bias in **Supplementary Fig. 14i**, which is around 10× faster than that of negative bias, indicating the excellent capability of the device to follow ultrafast switching light signals. We then measured the relative response with optical modulation. The 3 dB bandwidth of devices are extracted according to the dependence of photocurrent on the optical modulation frequency. As shown in **Supplementary Fig. 15**, the 3 dB cutoff frequency measured for device 4-6 can reach up to 130-195 kHz under 785 nm illumination.The response time of the device is estimated to be 2.7 μs -1.8 μs by the equation: $f_{3dB} = 0.35/t_r$, where $t_r$ is the response time of the device.

**Supplementary Table 1: Thickness parameters of heterojunction devices**

| Device \ Thickness (nm) | $WSe_2$ | $Ta_2NiSe_5$ |
|---|---|---|
| 1 | 60 | 13 |
| 2 | 69 | 17 |
| 3 | 35 | 23 |
| 4 | 13.5 | 32.9 |
| 5 | 10.1 | 11.6 |
| 6 | 6.9 | 24.1 |

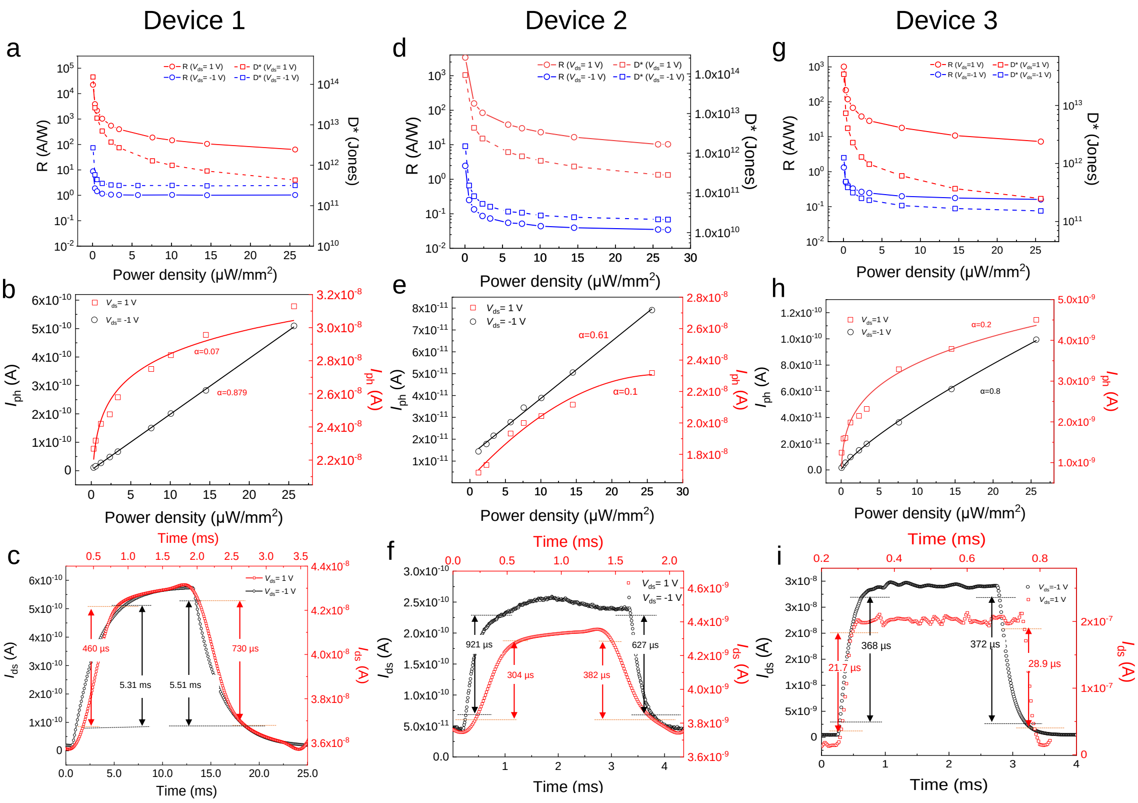


**Supplementary Fig. 13 Photoresponse of $WSe_2/Ta_2NiSe_5$ heterostructures with thicker $WSe_2$ (which is corresponding to devices 1, 2 and 3). a,d,g**, Responsivity and detectivity at

$V_{ds}$ = 1 V and -1 V under 785 nm. **b,e,h,** Photocurrent as a function of incident light power under 785 nm. **c,f,i,** Single magnified response curves under 785 nm.

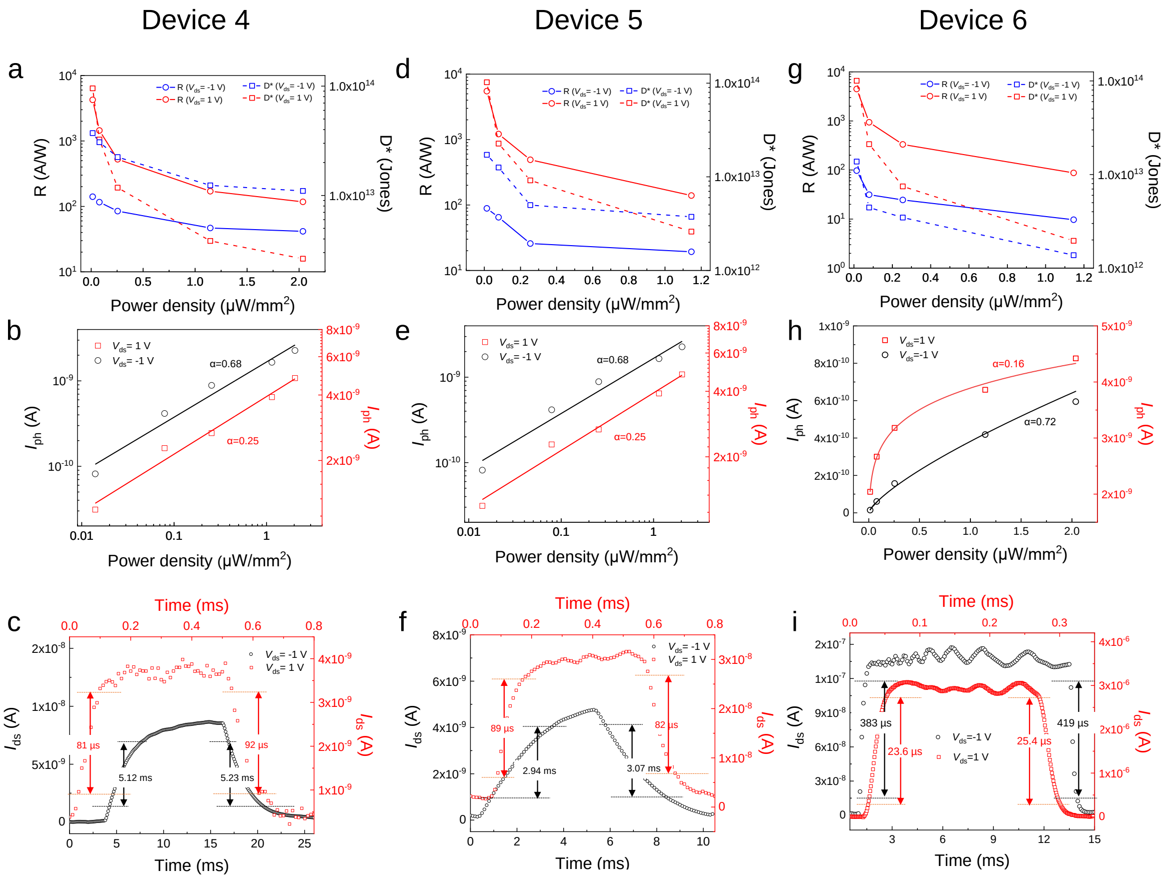


**Supplementary Fig. 14 Photoresponse of $WSe_2/Ta_2NiSe_5$ heterostructures with thinner $WSe_2$ (which is corresponding to devices 4, 5 and 6)**. **a,d,g,** Responsivity and detectivity at $V_{ds}$ = 1 V and -1 V under 785 nm. **b,e,h,** Photocurrent as a function of incident light power under 785 nm. **c, f, i,** Single magnified response curves under 785 nm.

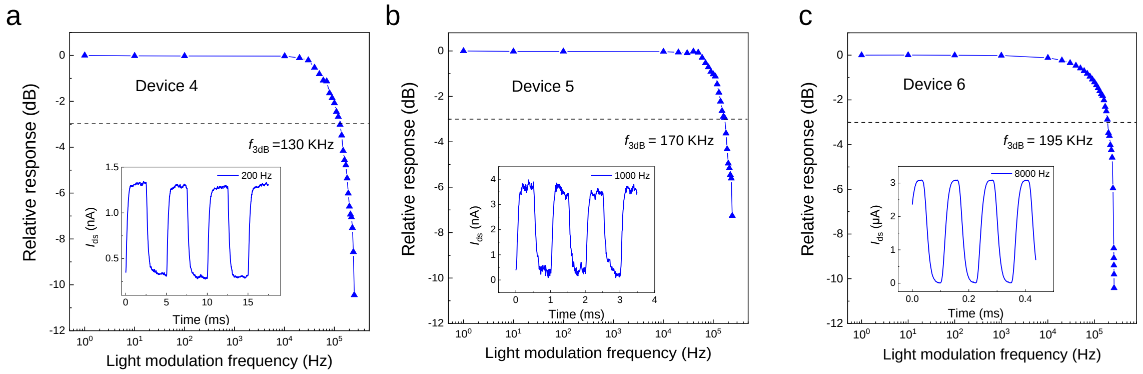


**Supplementary Fig. 15** Relative response with the modulation frequency measured for heterostructures with various thicknesses under 785 nm light illumination. The dashed line represents the relative response at 3 dB cutoff frequency. **Inset:** The time-dependent current of the devices.

## Supplementary Note 10. Device characterization under 532 and 633 nm illumination.

The optoelectronic response of the $WSe_2/Ta_2NiSe_5$ heterostructure device under 532 and 635 nm light irradiation were also investigated. The key parameters of the photodetector including responsivity, specific detectivity, and response time were assessed by measuring the photocurrent ($I_{ph}$) under the illuminated light with different powers. Here, we briefly discuss the photoelectric response characteristics of device 1 under 532 and 635 nm wavelength laser irradiation. The photodetection properties of the device at different bias voltages were depicted in **Supplementary Fig. 16**. As can be observed, simultaneously enhancement in responsivity and photoresponse speed under positive bias were also found under 532 and 635 nm as the case under 785 nm illumination in the main text.

**Supplementary Fig. 16a-c** show the device characteristics under 532 nm illumination. Based on the measured *I-V* curves at different powers, we calculated the performance parameters of the device under different $V_{ds}$. When $V_{ds}$ = -1 V, the device responsivity reaches a maximum of 8.21 A/W under 17.5 µW/mm$^2$ light power density, and the calculated value of detectivity is 5.74×10$^{11}$ Jones, which demonstrates good photodetection capability (**Supplementary Fig. 16a**). The photocurrent is then extracted and plotted as a function of light power in **Supplementary Fig. 16b**. The curve was fitted by the power-law equation of $I_{ph} \sim P^{\alpha}$, and we can see that the exponents $\alpha$ is ~0.89. As shown in **Supplementary Fig. 16c**, the rise and decay time were measured to be 3.13 ms and 5.76 ms, respectively. On stark contrary, at $V_{ds}$ = +1 V, the maximum responsivity achieved in the device is 610 A/W at the same power density, and the detectivity can reach 9.47×10$^{11}$ Jones. The light intensity dependent photocurrent can be estimated with $\alpha$ = 0.195, which is significantly smaller than the optimal value ($\alpha$ = 1), and indicates the participance of more traps/defects in the photodetector. Theoretically, if defect-induced photogating effect plays a dominant role, the response time would be longer. However, in our device, we found that the response time at positive bias is 353/557 µs, which is 10 times faster compared with the case under negative bias in **Supplementary Fig. 16c**.

**Supplementary Fig. 16d-f** show the device characteristics under 635 nm illumination. Similar to 532 and 785 nm, the device also has optimal photodetection capability under positive bias. As shown in **Supplementary Fig. 16d**, the maximum responsivity and detectivity obtained at $V_{ds}$ = +1 V are 397 A/W and 2.71×10$^{11}$ Jones under a power density of 0.56 µW/mm$^2$, respectively, which demonstrates excellent low light detection capability. The power dependence of the photocurrent was fitted with a $\alpha$ of 0.363 (**Supplementary Fig. 16e**). The

rise/fall times are 539/936 μs as shown in **Supplementary Fig. 16f**. Compared with the case at negative bias, the response speed is improved by nearly 10 times.

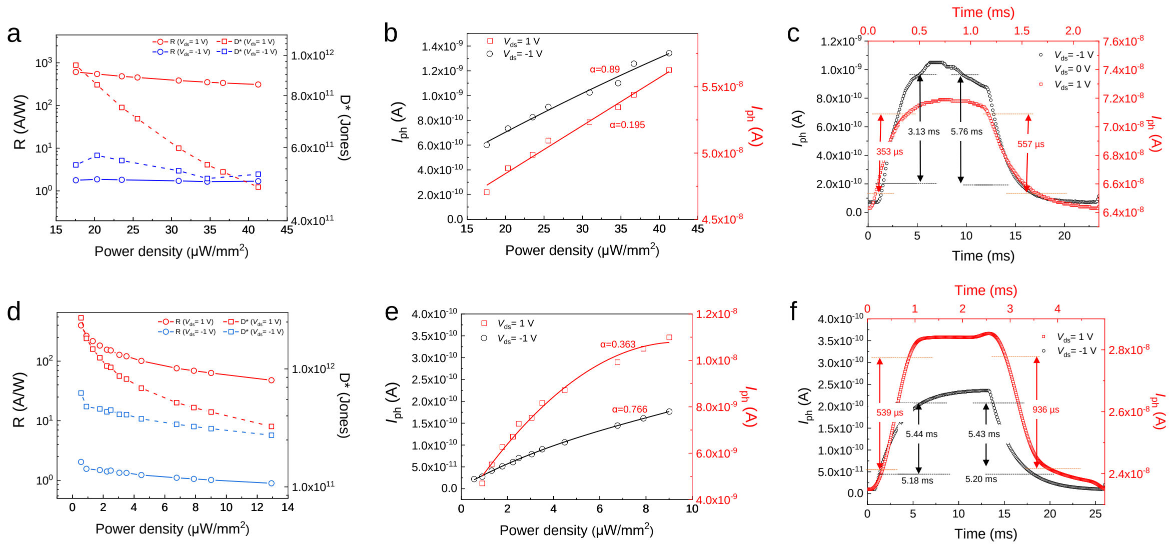

**Supplementary Fig. 16 Device characterization of device 1 under 532 nm and 633 nm illumination. a,** Responsivity and detectivity at $V_{ds}$ = -1 V and 1 V under 532 nm. **b,** Photocurrent as a function of incident light power under 532 nm. **c,** Single magnified response curve under 532 nm. **d,** Responsivity and detectivity at $V_{ds}$ = -1 V and 1 V under 633 nm. **e,** Photocurrent as a function of incident light power under 633 nm. **f,** Single magnified response curve under 633 nm.

## Supplementary Note 11. Device noise and specific detectivity.

As the key figures of merit of photodetectors, the noise and specific detectivity are also characterized. To calculate these values, the time-resolved dark currents with applying a constant drain bias ($V_{ds}$) at -1 V, 0 V and 1 V, have been measured (**Supplementary Fig. 17a-c**). By taking the Fourier transform of dark current traces, the noise spectral densities ($S_n$) as a function of frequency are obtained as shown in **Supplementary Fig. 17d** ($V_{ds}$ = -1 V), **Supplementary Fig. 17e** ($V_{ds}$ = 0 V) and **Supplementary Fig. 17f** ($V_{ds}$ = 1 V).[29-31]

Theoretically, the noise current consists of several parts that can be expressed as:[32]

$$i_{noise} = \sqrt{{i_{shot}}^2 + {i_{thermal}}^2 + {i_{1/f}}^2 + {i_{g-r}}^2}$$

$$= \sqrt{2eI_dB + \frac{4KBT}{R_\Omega} + {i(f,B)_{1/f}}^2 + {i(f,B)_{g-r}}^2} \quad (1)$$

where $I_d$ is the dark current, $e$ is the elementary charge, $B$ is the bandwidth, $T$ is the temperature, and $R_\Omega$ is the shunt resistance of device (The value of $R_\Omega$ can be obtained by

fitting the *I-V* experimental data under weak voltage). The $i_{thermal}$ is thermal noise, $i_{shot}$ is shot noise, $i_{1/f}$ is *1/f* noise, and $i_{g-r}$ is generation-recombination noise, respectively. The *1/f* noise and *g-r* noise are dominant at low frequencies, ascribing to the interface traps or defects.[30, 33] As is shown in **Supplementary Fig. 17d**,**e**, when a bias voltage of -1 V and 0 V are applied, since the noise spectrum is almost frequency independent at the bandwidth of 1 Hz, white noise is the primary source of noise current. Therefore, the noise current ($i_{noise}$) can be expressed as:

$$i_{noise} = \sqrt{2eI_dB + \frac{4KBT}{R_\Omega}} \quad (2)$$

where $\sqrt{2eI_dB}$ is short noise and $\sqrt{\frac{4KBT}{R_\Omega}}$ is thermal noise. Due to the ultra-large value of $R_\Omega$ in our device ($R_\Omega > 7.9$ GΩ), the ${i_{thermal}}^2$ is far less than ${i_{shot}}^2$ (${i_{thermal}}^2 \ll (i_{shot}/10)^2$), so the noise current can be estimated as $i_{noise} = \sqrt{2eI_dB}$. It is obvious that the noise mainly comes from the dark current. According to the noise spectral density extracted at the bandwidth of 1 Hz, the calculated *D** for Device 1 as listed in **Supplementary Note 9** is $2.18\times10^{12}$ Jones ($V_{ds}$ = -1 V) and $9.6\times10^{10}$ Jones ($V_{ds}$ = 0 V) at 785 nm with a power density of 0.05 μW/mm$^2$, respectively.

However, under the bias voltage of +1 V, we found that the noise current has a weak frequency dependence at around the bandwidth of 1 Hz. Thus, the specific detectivity can be expressed as:[29]

$$D^* = \frac{R\sqrt{A}}{S_n} \quad (3)$$

where *R* is responsivity and *A* is device active area. According to the formula (3), the *D** is $8.3\times10^{13}$ Jones at 785 nm for Device 1 with a power density of 0.05 μW/mm$^2$, which is very close to the detectivity ($1.5\times10^{14}$ Jones) calculated assuming the dark current dominants the noise current. Thereby, to simplified, we derived the specific detectivity from the formula $D^* = RA^{1/2}/(2qI_{dark})^{1/2}$ for the Device 1 to 6 as listed in above **Supplementary Note 9**.

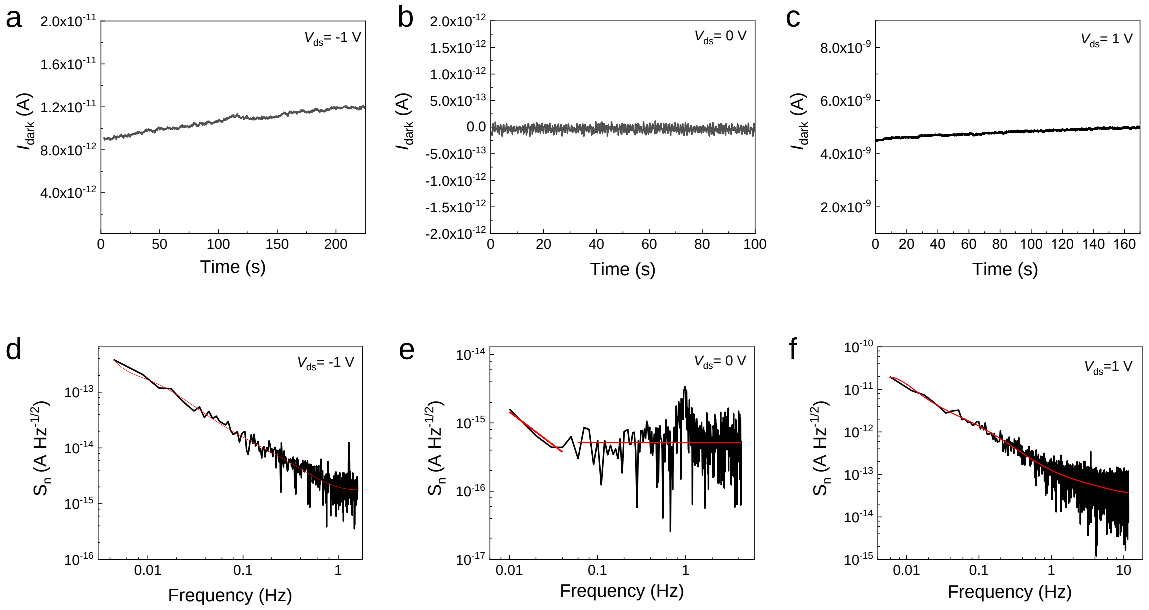


**Supplementary Fig. 17 Device noise and specific detectivity. a-c**, The time-resolved dark currents at (a) $V_{ds}$ = -1 V, (b) $V_{ds}$ = 0 V and (c) $V_{ds}$ = 1 V. **d-f**, The noise spectral densities ($S_n$) as a function of frequency at (d) $V_{ds}$ = -1 V, (e) $V_{ds}$ = 0 V and (f) $V_{ds}$ = 1 V.

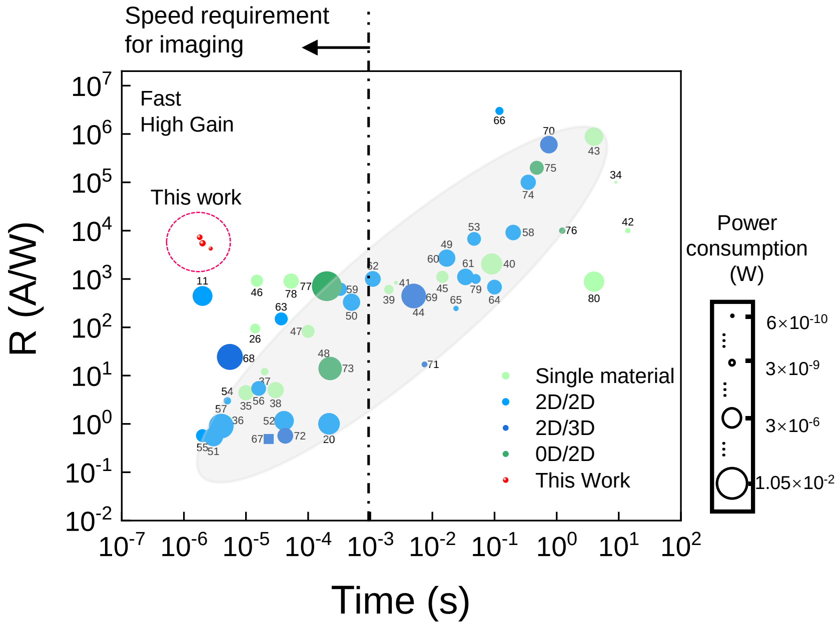


**Supplementary Fig. 18 Comparison of device responsivity and response time with previous reports (grey ellipsoid).**[11, 20, 26, 34-80] The $WSe_2/Ta_2NiSe_5$ heterostructures with different thicknesses as described in the main text and Supplementary Note 9 are included. The measured power consumption are 1.54 nW, 5.14 nW and 3.73 nW for different thicknesses, respectively.

## Supplementary Note 12. Broadband infrared photoresponse of the $WSe_2/Ta_2NiSe_5$ heterostructure.

**Supplementary Fig. 19** shows the photoelectric response of the device under the illumination of infrared light. The corresponding current−voltage ($I_{ds}$−$V_{ds}$) curves measured both in dark and under 1064 nm to 2200 nm light illuminations are demonstrated in **Supplementary Fig. 19a-d**. The device shows typical junction characteristics as seen from the figures. At positive bias voltage, the current is gradually enhanced with the applied voltage ($V_{ds}$) because of the increase of carrier density. Furthermore, we found that the device can operate at zero $V_{ds}$, showing its self-powered operation capability. **Supplementary Fig. 19e-h** depicts the time-dependent photoresponse of the $WSe_2/Ta_2NiSe_5$ heterostructure device ($V_{ds}$ = 1 V, $V_{gs}$ = 0 V). Apparently, the device shows stable responses to the fast-varying light signals under different light powers.

The parameters of device performance were further derived according the results in **Supplementary Fig. 19**. When +1 V bias voltage ($V_{ds}$ = 1 V) is applied, the $R$ and $D^*$ were calculated to be 830 A/W and $5.6\times10^{12}$ Jones under light intensity of 0.002 mW/mm$^2$ at 1064 nm. Both $R$ and $D^*$ are found to decrease with increasing power intensity (**Supplementary Fig. 20a**). As illustrated in **Supplementary Fig. 20b**, the dependence of photocurrent on light intensity can be well fitted with a power exponent $\alpha$ of 0.028 ($\alpha < 1$) at 1064 nm, which indicates the presence of traps and defects in the photodetector.[54] In addition, the rise/fall time is determined to be 459/782 μs from the single magnified response curve in **Supplementary Fig. 20c**, and the response speed is increased by about 10 times as compared with the case at $V_{ds}$ = -1 V (see **Supplementary Fig. 20f**). Similarly, **Supplementary Fig. 20a** shows the maximum responsivity of 334.7 A/W (@0.009 mW/mm$^2$) and 79.8 A/W (@0.005 mW/mm$^2$) can be achieved under 1310 and 1550 nm. Meantime, the corresponding $D^*$ is evaluated as $2\times10^{12}$ Jones at 1310 nm and $5\times10^{11}$ Jones at 1550 nm, respectively. In addition, according to the power-dependent photocurrent, the fitted $\alpha$ is 0.02 and 0.05 at 1310 nm and 1550 nm, respectively (**Supplementary Fig. 20b**). The photoresponse speed is calculated with a rise/fall time of 486/585 μs at 1310 nm and 520/634 μs at 1550 nm. Even under the illumination of 2200 nm wavelength, the $R$ and $D^*$ can reach 2.78 A/W and $1.9\times10^{10}$ Jones (@0.106 mW/mm$^2$), respectively (**Supplementary Fig. 20a**), which are comparable to the state of the commercial photodetectors.[81-83] Moreover, the response time of the device was extracted to be 80.1 ms/90.4 ms in **Supplementary Fig. 20c**.

**Supplementary Fig. 20d-f** show the optoelectronic response of the device under negative bias ($V_{ds}$= -1 V). Under the illumination of 1064 nm light, the $R$ of the device is 368 mA/W under light illumination of 0.002 mW/mm$^2$ (**Supplementary Fig. 20d**), which is three orders of magnitude less than that under forward bias. The corresponding $D*$ is 1.12 × $10^{11}$ Jones. The device has a longer response time with a rise/fall time of 5.11/5.55 ms (**Supplementary Fig. 20f**). Compared with the cases under positive bias voltage, the response speed is reduced by about 10 times.

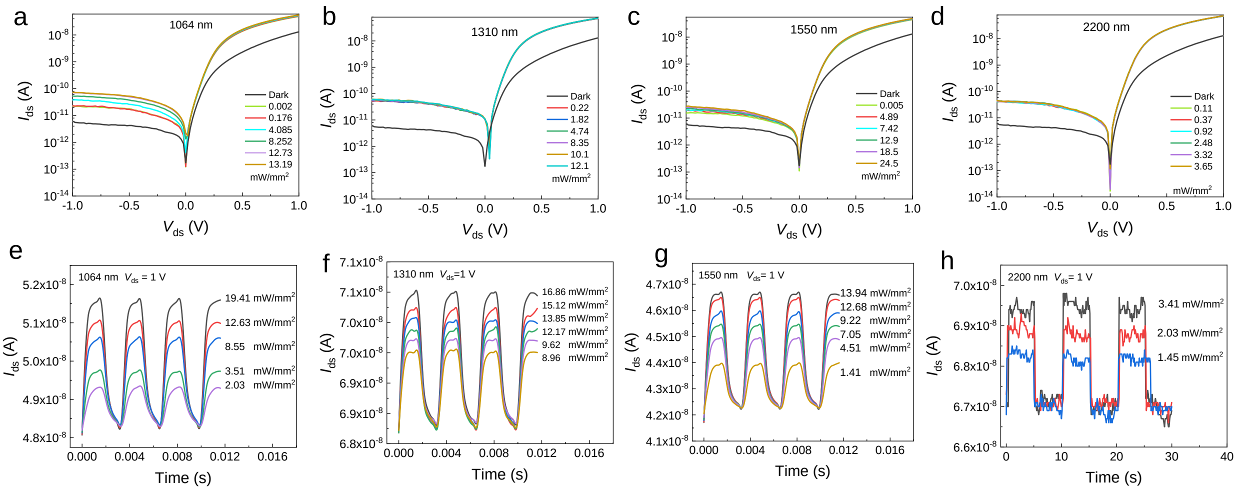


**Supplementary Fig. 19 Broadband infrared photoresponse of the $WSe_2/Ta_2NiSe_5$ heterostructure. a-d**, The current−voltage ($I_{ds}$−$V_{ds}$) curves measured both in dark and under 1064 nm to 2200 nm light illuminations. **e-h**, The time-dependent photoresponse under 1064 nm to 2200 nm light illuminations. $V_{ds}$ = 1 V, $V_{gs}$ = 0 V.

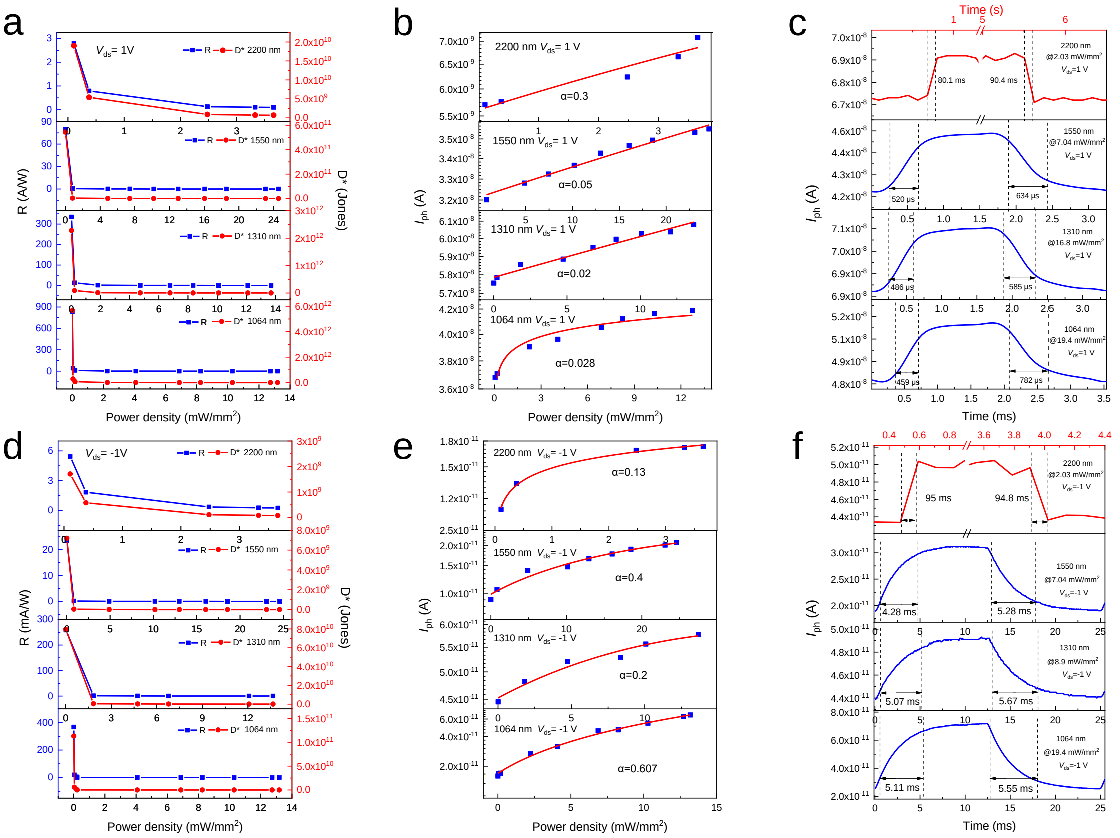


**Supplementary Fig. 20 Device characterization under different light illumination at $V_{ds}$ = 1 V and -1 V. a,** Responsivity and detectivity at 1064 nm, 1310 nm, 1550 nm, 2200 nm at $V_{ds}$ = 1 V. **b**, Photocurrent as a function of incident light powers at $V_{ds}$ = 1 V. **c**, Single magnified response curves at $V_{ds}$ = 1 V. **d,** Responsivity and detectivity at 1064 nm, 1310 nm, 1550 nm, 2200 nm at $V_{ds}$ = -1 V. **e**, Photocurrent as a function of incident light powers at $V_{ds}$ = -1 V. **f**, Single magnified response curves at $V_{ds}$ = -1 V.

## Supplementary Note 13. EQE of the $WSe_2/Ta_2NiSe_5$ heterostructure.

The EQE of a photodetector represents the number of collected charge carriers per incident photon. It can be described using the formula EQE = $I_{ph}/P_{in}$ ($\hbar c/e\lambda$), where $I_{ph}$, $\hbar$, $c$, $e$, $\lambda$ and $P_{in}$ denote the photocurrent, Planck constant, the speed of light, the electronic charge, the incident wavelength and incident light power, respectively. The $I_{ph}$ and EQE values at different light wavelengths under $V_{ds}$ = 1 V are plotted in **Supplementary Fig. 21**. The maximum EQE values is calculated to be around $3.51\times10^6$ % at 785 nm under the light intensity of 0.05 μW/mm$^2$. Note that the $I_{ph}$ is proportional to the incident light power, but EQE values decrease with increasing light intensity, and this phenomenon may originate from the increase in the charge carrier scattering and the rate of charge recombination.[84] In addition, we found that the EQE of the device is significantly larger than 100%, which verifies that the device has obvious optical gain.[85, 86] The EQE is estimated to be about $3.23\times10^4$ % at 532 nm (@17.55 μW/mm$^2$), $7.75\times10^4$ % at 635 nm (@0.56 μW/mm$^2$). In the infrared range, the device still has high EQE, and the maximum EQE obtained is $9.68\times10^4$ % under 1064 nm illumination at a power density of 0.002 mW/mm$^2$. The EQE values are $3\times10^4$ % (@1310 nm, 0.009 mW/mm$^2$) and $9.5\times10^3$ % (@1550 nm, 0.005 mW/mm$^2$), respectively. The EQE of the device decreases to 157% (0.106 mW/mm$^2$) at 2200 nm. Compared with the visible band, the decrease of EQE is mainly due to the relatively weak light absorption of the device at longer wavelengths.

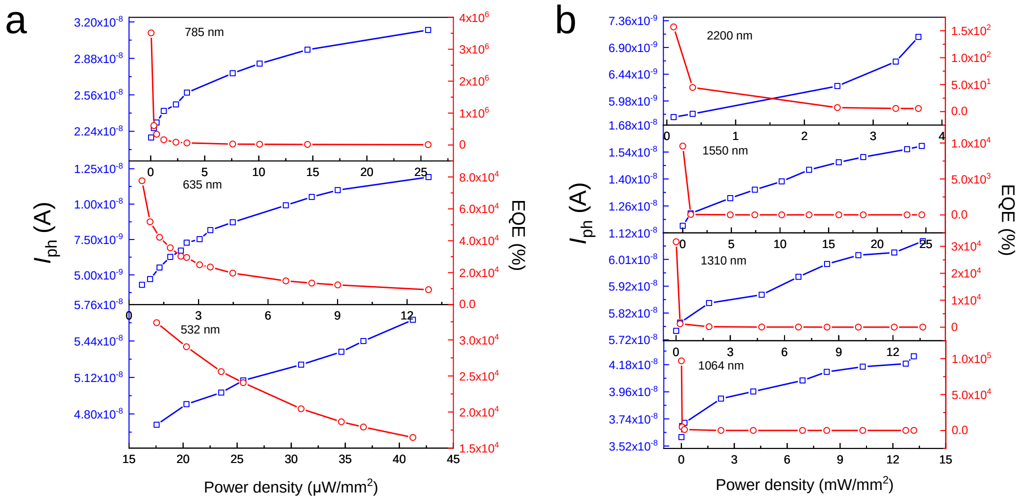


**Supplementary Fig. 21 EQE as a function of incident light power at different wavelengths of a**, 532 nm. 635 nm and 785 nm. **b**, 1064 nm, 1310 nm, 1550 nm and 2200 nm.

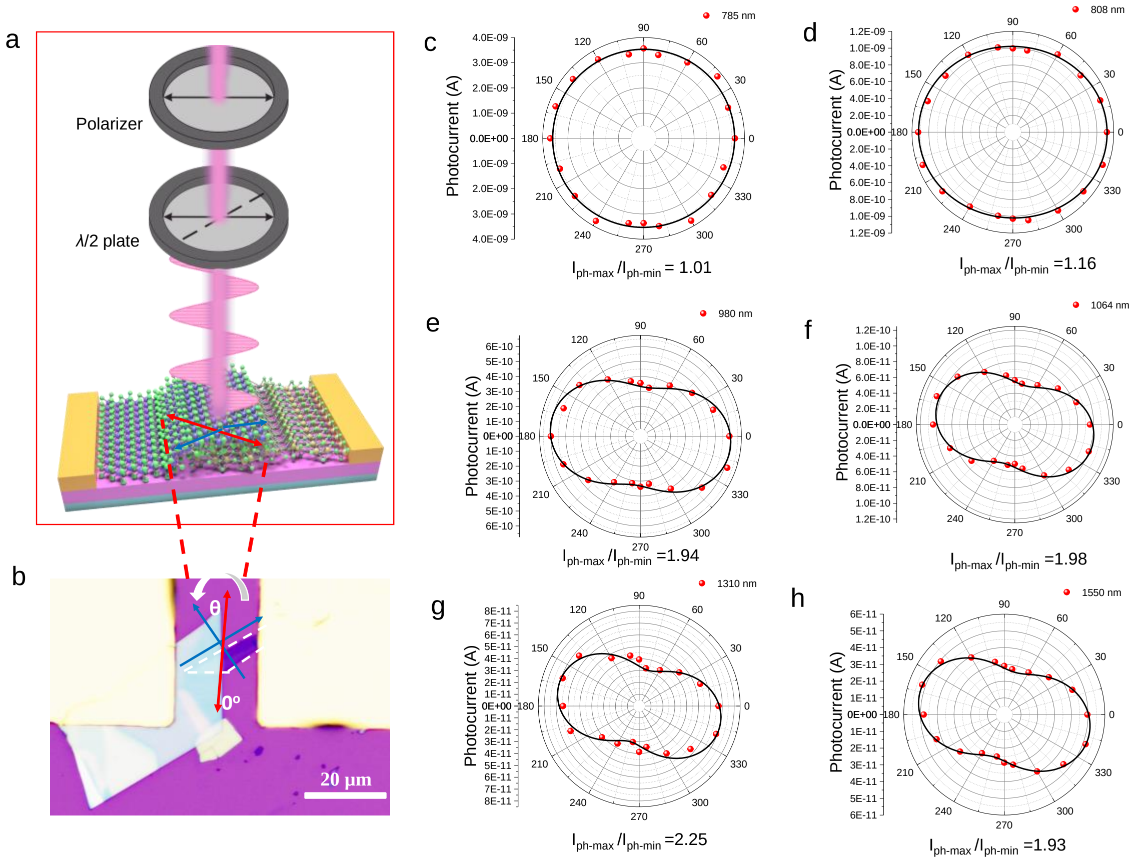


**Supplementary Fig. 22 Polarized photoresponse of the $WSe_2/Ta_2NiSe_5$ heterostructure**. **a-b**, Schematic light path and measurement configuration of device polarized photoresponse. The red and white arrows represent the direction of onset and rotation of the polarized light, respectively, and the angle of rotation is defined as $\theta$. **c-h**, The polar plots of polarized photocurrents under illumination of different wavelengths from 785 to 1550 nm at $V_{ds}$ = -1 V.

When visible light irradiates on the device (λ≤808 nm), the anisotropy in photocurrent ($I_{ph\text{-}max}/I_{ph\text{-}min}$) is quite small, implying the less sensitivity to visible polarized light. This phenomenon indicates that the bandgap absorption of the $WSe_2$ dominates the heterostructure overall absorption at shorter wavelengths. However, under infrared light illumination (λ >808 nm), more visibly wavelength-dependent polarized photoresponse is observed, which are mainly attributed to the anisotropic absorption of $Ta_2NiSe_5$ at longer wavelengths.

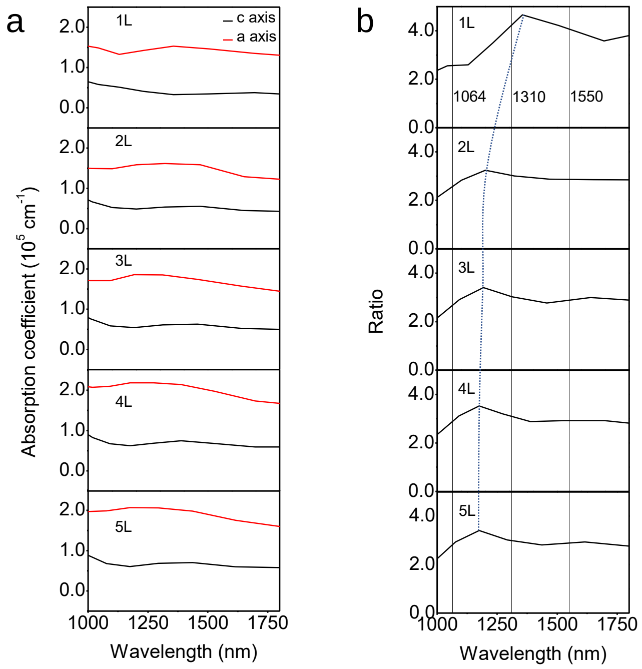


**Supplementary Fig. 23 Calculated optical absorption coefficients of monolayer (1L) to five-layer (5L) $Ta_2NiSe_5$. a**, Optical absorption coefficient of $Ta_2NiSe_5$ along *a* axis and *c* axis, respectively. **b**, The anisotropy ratio of optical absorption coefficients between *a* and *c* directions. The left shift of wavelength for the maximum *a*/*c* ratio from monolayer to multilayer $Ta_2NiSe_5$ is likely due to the coupling effect between different layers.[87, 88]

## Supplementary Note 14. Bias-tunable anisotropic ratio of the $WSe_2/Ta_2NiSe_5$ heterostructure photodetector.

The anisotropic ratio β in our device is bias-dependent, *i.e.* larger β is obtained under negative $V_{ds}$ than under positive $V_{ds}$. Under negative $V_{ds}$, the device behaves like a photoconductor, in which the polarized photoresponse is directly induced by the intrinsic anisotropic absorption of $Ta_2NiSe_5$. However, under positive $V_{ds}$, the polarized photoresponse of the device is a more complex process, which can be explained as follows. First, $Ta_2NiSe_5$ generates photocarriers under polarized light illumination, and the photoexcited electrons are blocked at the $WSe_2/Ta_2NiSe_5$ interface, inducing a photogating effect; Then, the photogating leads to the change of Fermi level of $WSe_2$ and increment of the hole concentration; Finally, the change of hole concentration further tunes the tunneling barrier at $WSe_2$/metal contact, and photogating-assisted carrier tunneling occurs, resulting in a fast carrier transit time under positive biases. We conclude that the anisotropic photoresponse under positive biases is an indirect result of anisotropic absorption of $Ta_2NiSe_5$. During the above multi-processes, the scattering or loss of the anisotropic photocarriers may be increased and thus weakens the anisotropic response of the device.

# Supplementary References


1. Jog H., Harnagea L., Mele Eugene J., Agarwal R. Exchange coupling–mediated broken symmetries in $Ta_2NiSe_5$ revealed from quadrupolar circular photogalvanic effect. *Sci. Adv.* **8**, eabl9020 (2022).

2. Zhang Y.-S. a. B., Jan A. N. and Matsumoto, Yosuke and Isobe, Masahiko and Takagi, Hidenori. Thermal transport signatures of the excitonic transition and associated phonon softening in the layered chalcogenide $Ta_2NiSe_5$. *Phys. Rev. B* **104**, L121201 (2021).

3. Qiao J., Feng F., Song S., Wang T., Shen M., Zhang G., *et al.* Perovskite quantum dot-$Ta_2NiSe_5$ mixed-dimensional van der Waals heterostructures for high-performance near-infrared photodetection. *Adv. Funct. Mater.* **32**, 2110706 (2022).

4. Kim K., Kim H., Kim J., Kwon C., Kim J. S., Kim B. J. Direct observation of excitonic instability in $Ta_2NiSe_5$. *Nat. Commun.* **12**, 1969 (2021).

5. Li L., Wang W., Gan L., Zhou N., Zhu X., Zhang Q., *et al.* Ternary $Ta_2NiSe_5$ flakes for a high-performance infrared photodetector. *Adv. Funct. Mater.* **26**, 8281-8289 (2016).

6. Liu Y., Li H., Qiu C., Hu X., Liu D. Layer-dependent signatures for exciton dynamics in monolayer and multilayer $WSe_2$ revealed by fluorescence lifetime imaging measurement. *Nano Res.* **13**, 661-666 (2020).

7. Zhao W., Ghorannevis Z., Chu L., Toh M., Kloc C., Tan P.-H., *et al.* Evolution of electronic structure in atomically thin sheets of $WS_2$ and $WSe_2$. *ACS Nano* **7**, 791-797 (2013).

8. Wang G., Li L., Fan W., Wang R., Zhou S., Lü J.-T., *et al.* Interlayer coupling induced infrared response in $WS_2/MoS_2$ heterostructures enhanced by surface plasmon resonance. *Adv. Funct. Mater.* **28**, 1800339 (2018).

9. Wu H., Kang Z., Zhang Z., Zhang Z., Si H., Liao Q., *et al.* Interfacial charge behavior modulation in perovskite quantum dot-monolayer $MoS_2$ 0D-2D mixed-dimensional van der Waals heterostructures. *Adv. Funct. Mater.* **28**, 1802015 (2018).

10. Wu H., Si H., Zhang Z., Kang Z., Wu P., Zhou L., *et al.* All-Inorganic perovskite quantum dot-monolayer $MoS_2$ mixed-dimensional van der Waals heterostructure for ultrasensitive photodetector. *Adv. Sci.* **5**, 1801219 (2018).

11. Luo P., Wang F., Qu J., Liu K., Hu X., Liu K., *et al.* Self-Driven $WSe_2/Bi_2O_2Se$ van der Waals heterostructure photodetectors with high light on/off ratio and fast response. *Adv. Funct. Mater.* **31**, 2008351 (2021).

12. Mak K. F., Lee C., Hone J., Shan J., Heinz T. F. Atomically thin $MoS_2$: a new direct-gap semiconductor. *Phy. Rev. Lett.* **105**, 136805 (2010).

13. Zhu X., Li F., Wang Y., Qiao M., Li Y. $Pd_2Se_3$ monolayer: a novel two-dimensional material with excellent electronic, transport, and optical properties. *J. Mater. Chem. C.* **6**, 4494-4500 (2018).

14. Sun Y., Luo S., Zhao X.-G., Biswas K., Li S.-L., Zhang L. InSe: a two-dimensional material with strong interlayer coupling. *Nanoscale* **10**, 7991-7998 (2018).

15. Lv L., Yu J., Hu M., Yin S., Zhuge F., Ma Y., *et al.* Design and tailoring of two-dimensional Schottky, pn and tunnelling junctions for electronics and optoelectronics. *Nanoscale* **13**, 6713-6751 (2021).

16. Ramadan W., Ogale S. B., Dhar S., Fu L. F., Shinde S. R., Kundaliya D. C., *et al.* Electrical properties of epitaxial junctions between Nb:$SrTiO_3$ and optimally doped, underdoped, and Zn-doped $YBa_2Cu_3O_{7-\delta}$. *Phys. Rev. B.* **72**, 205333 (2005).

17. Lee K., Kim Y., Kim D., Lee J., Lee H., Joo M.-K., *et al.* Metal-contact improvement in a multilayer $WSe_2$ transistor through strong hot carrier injection. *ACS Appl. Mater. Interfaces* **13**, 2829-2835 (2021).

18. Smyth C. M., Walsh L. A., Bolshakov P., Catalano M., Addou R., Wang L., *et al.* Engineering the Palladium–$WSe_2$ interface chemistry for field effect transistors with high-performance hole contacts. *ACS Appl. Nano Mater.* **2**, 75-88 (2019).

19. Zakirov E. R., Kesler V. G. The Gaussian distribution of barrier heights in Au/native fluoride/nHgCdTe Schottky diodes. 2016 17th International Conference of Young Specialists on Micro/Nanotechnologies and Electron Devices (EDM); 2016 30 June-4 July 2016; 2016. p. 11-15.

20. Wu F., Xia H., Sun H., Zhang J., Gong F., Wang Z., *et al.* AsP/InSe van der Waals tunneling heterojunctions with ultrahigh reverse rectification ratio and high photosensitivity. *Adv. Funct. Mater.* **29**, 1900314 (2019).

21. Mukherjee B., Hayakawa R., Watanabe K., Taniguchi T., Nakaharai S., Wakayama Y. $ReS_2$/h-BN/Graphene heterostructure based multifunctional devices: tunneling diodes, FETs, logic gates, and memory. *Adv. Electron. Mater.* **7**, 2000925 (2021).

22. Sarkar D., Xie X., Liu W., Cao W., Kang J., Gong Y., *et al.* A subthermionic tunnel field-effect transistor with an atomically thin channel. *Nature* **526**, 91-95 (2015).

23. Doan M.-H., Jin Y., Adhikari S., Lee S., Zhao J., Lim S. C., *et al.* Charge transport in $MoS_2$/$WSe_2$ van der Waals heterostructure with tunable inversion layer. *ACS Nano* **11**, 3832-3840 (2017).

24. Guo D. Y., Wu Z. P., An Y. H., Guo X. C., Chu X. L., Sun C. L., *et al.* Oxygen vacancy tuned Ohmic-Schottky conversion for enhanced performance in β-$Ga_2O_3$ solar-blind ultraviolet photodetectors. *Appl. Phys. Lett.* **105**, 023507 (2014).

25. Guo D., Su Y., Shi H., Li P., Zhao N., Ye J., *et al.* Self-Powered ultraviolet photodetector with superhigh photoresponsivity (3.05 A/W) based on the GaN/Sn:$Ga_2O_3$ pn Junction. *ACS Nano* **12**, 12827-12835 (2018).

26. Ghosh S., Varghese A., Thakar K., Dhara S., Lodha S. Enhanced responsivity and detectivity of fast $WSe_2$ phototransistor using electrostatically tunable in-plane lateral p-n homojunction. *Nat. Commun.* **12**, 3336 (2021).

27. Feng S., Liu C., Zhu Q., Su X., Qian W., Sun Y., *et al.* An ultrasensitive molybdenum-based double-heterojunction phototransistor. *Nat. Commun.* **12**, 4094 (2021).

28. Zhang B. Y., Liu T., Meng B., Li X., Liang G., Hu X., *et al.* Broadband high photoresponse from pure monolayer graphene photodetector. *Nat. Commun.* **4**, 1811 (2013).

29. Nikitskiy I., Goossens S., Kufer D., Lasanta T., Navickaite G., Koppens F. H. L., *et al.* Integrating an electrically active colloidal quantum dot photodiode with a graphene phototransistor. *Nat. Commun.* **7**, 11954 (2016).

30. Xiong J., Sun Y., Wu L., Wang W., Gao W., Huo N., *et al.* High performance self-driven polarization-sensitive photodetectors based on GeAs/InSe heterojunction. *Adv. Opt. Mater.* **9**, 2101017 (2021).

31. Han X., Wen P., Zhang L., Gao W., Chen H., Gao F., *et al.* A polarization-sensitive self-powered photodetector based on a p-$WSe_2$/$TaIrTe_4$/n-$MoS_2$ van der Waals heterojunction. *ACS Appl. Mater. Interfaces* **13**, 61544-61554 (2021).

32. Jansen-van Vuuren R. D., Armin A., Pandey A. K., Burn P. L., Meredith P. Organic photodiodes: the future of full color detection and image sensing. *Adv. Mater.* **28**, 4766-4802 (2016).

33. Huo N., Gupta S., Konstantatos G. $MoS_2$–HgTe quantum dot hybrid photodetectors beyond 2 µm. *Adv. Mater.* **29**, 1606576 (2017).

34. Island J. O., Blanter S. I., Buscema M., van der Zant H. S. J., Castellanos-Gomez A. Gate controlled photocurrent generation mechanisms in high-gain $In_2Se_3$ phototransistors. *Nano Lett.* **15**, 7853-7858 (2015).

35. Chen G., Yu Y., Zheng K., Ding T., Wang W., Jiang Y., *et al.* Fabrication of ultrathin $Bi_2S_3$ nanosheets for high-performance, flexible, Visible–NIR photodetectors. *Small* **11**, 2848-2855 (2015).

36. Yu S. H., Lee Y., Jang S. K., Kang J., Jeon J., Lee C., *et al.* Dye-sensitized $MoS_2$ photodetector with enhanced spectral photoresponse. *ACS Nano* **8**, 8285-8291 (2014).

37. Lv L., Zhuge F., Xie F., Xiong X., Zhang Q., Zhang N., *et al.* Reconfigurable two-dimensional optoelectronic devices enabled by local ferroelectric polarization. *Nat. Commun.* **10**, 3331 (2019).

38. Wu G., Wang X., Chen Y., Wu S., Wu B., Jiang Y., *et al.* $MoTe_2$ p–n homojunctions defined by ferroelectric polarization. *Adv. Mater.* **32**, 1907937 (2020).

39. Hafeez M., Gan L., Li H., Ma Y., Zhai T. Large-area bilayer $ReS_2$ film/multilayer $ReS_2$ flakes synthesized by chemical vapor deposition for high performance photodetectors. *Adv. Funct. Mater.* **26**, 4551-4560 (2016).

40. Wang J., Lian G., Xu Z., Fu C., Lin Z., Li L., *et al.* Growth of large-size SnS thin crystals driven by oriented attachment and applications to gas sensors and photodetectors. *ACS Appl. Mater. Interfaces* **8**, 9545-9551 (2016).

41. Lian Q., Zhu X., Wang X., Bai W., Yang J., Zhang Y., *et al.* Ultrahigh-detectivity photodetectors with van der Waals epitaxial CdTe single-crystalline films. *Small* **15**, 1900236 (2019).

42. Jang J., Lee Y., Yoon J.-Y., Yoon H. H., Koo J., Choe J., *et al.* One-dimensional assembly on two-dimensions: AuCN nanowire epitaxy on graphene for hybrid phototransistors. *Nano Lett.* **18**, 6214-6221 (2018).

43. Liu E., Long M., Zeng J., Luo W., Wang Y., Pan Y., *et al.* High responsivity phototransistors based on few-layer $ReS_2$ for weak signal detection. *Adv. Funct. Mater.* **26**, 1938-1944 (2016).

44. Zhou J., Zeng Q., Lv D., Sun L., Niu L., Fu W., *et al.* Controlled synthesis of high-quality monolayered $\alpha$-$In_2Se_3$ via physical vapor deposition. *Nano Lett.* **15**, 6400-6405 (2015).

45. Zhou X., Gan L., Tian W., Zhang Q., Jin S., Li H., *et al.* Ultrathin $SnSe_2$ flakes grown by chemical vapor deposition for high-performance photodetectors. *Adv. Mater.* **27**, 8035-8041 (2015).

46. Krishnamurthi V., Khan H., Ahmed T., Zavabeti A., Tawfik S. A., Jain S. K., *et al.* Liquid-metal synthesized ultrathin SnS layers for high-performance broadband photodetectors. *Adv. Mater.* **32**, 2004247 (2020).

47. Guo Q., Pospischil A., Bhuiyan M., Jiang H., Tian H., Farmer D., *et al.* Black phosphorus mid-infrared photodetectors with high gain. *Nano Lett.* **16**, 4648-4655 (2016).

48. Liu H., Zhu X., Sun X., Zhu C., Huang W., Zhang X., *et al.* Self-powered broad-band photodetectors based on vertically stacked $WSe_2/Bi_2Te_3$ p–n heterojunctions. *ACS Nano* **13**, 13573-13580 (2019).

49. Shin G. H., Park C., Lee K. J., Jin H. J., Choi S.-Y. Ultrasensitive phototransistor based on $WSe_2$–$MoS_2$ van der Waals heterojunction. *Nano Lett.* **20**, 5741-5748 (2020).

50. Liu H., Li D., Ma C., Zhang X., Sun X., Zhu C., *et al.* Van der Waals epitaxial growth of vertically stacked $Sb_2Te_3/MoS_2$ p–n heterojunctions for high performance optoelectronics. *Nano Energy* **59**, 66-74 (2019).

51. Ahn J., Kyhm J.-H., Kang H. K., Kwon N., Kim H.-K., Park S., *et al.* 2D $MoTe_2/ReS_2$ van der Waals heterostructure for high-performance and linear polarization-sensitive photodetector. *ACS Photonics* **8**, 2650-2658 (2021).

52. Zhong J., Wu B., Madoune Y., Wang Y., Liu Z., Liu Y. $PdSe_2/MoSe_2$ vertical heterojunction for self-powered photodetector with high performance. *Nano Res.* **15**, 2489-2496 (2022).

53. Ye K., Liu L., Liu Y., Nie A., Zhai K., Xiang J., *et al.* Lateral bilayer $MoS_2$–$WS_2$ heterostructure photodetectors with high responsivity and detectivity. *Adv. Opt. Mater.* **7**, 1900815 (2019).

54. Varghese A., Saha D., Thakar K., Jindal V., Ghosh S., Medhekar N. V., *et al.* Near-Direct bandgap $WSe_2$/$ReS_2$ Type-II pn heterojunction for enhanced ultrafast photodetection and high-performance photovoltaics. *Nano Lett.* **20**, 1707-1717 (2020).

55. Ahn J., Ko K., Kyhm J.-h., Ra H.-S., Bae H., Hong S., *et al.* Near-Infrared self-powered linearly polarized photodetection and digital incoherent holography using $WSe_2$/$ReSe_2$ van der Waals heterostructure. *ACS Nano* **15**, 17917-17925 (2021).

56. Lee J.-B., Lim Y. R., Katiyar A. K., Song W., Lim J., Bae S., *et al.* Direct synthesis of a self-assembled $WSe_2$/$MoS_2$ heterostructure array and its optoelectrical properties. *Adv. Mater.* **31**, 1904194 (2019).

57. Bullock J., Amani M., Cho J., Chen Y.-Z., Ahn G. H., Adinolfi V., *et al.* Polarization-resolved black phosphorus/molybdenum disulfide mid-wave infrared photodiodes with high detectivity at room temperature. *Nat. Photon.* **12**, 601-607 (2018).

58. Zhou X., Zhou N., Li C., Song H., Zhang Q., Hu X., *et al.* Vertical heterostructures based on $SnSe_2$/$MoS_2$ for high performance photodetectors. *2D Mater.* **4**, 025048 (2017).

59. Heo J., Jeong H., Cho Y., Lee J., Lee K., Nam S., *et al.* Reconfigurable van der Waals heterostructured devices with metal–insulator transition. *Nano Lett.* **16**, 6746-6754 (2016).

60. Li A., Chen Q., Wang P., Gan Y., Qi T., Wang P., *et al.* Ultrahigh-sensitive broadband photodetectors based on dielectric shielded $MoTe_2$/Graphene/$SnS_2$ p–g–n Junctions. *Adv. Mater.* **31**, 1805656 (2019).

61. Fang C., Wang H., Shen Z., Shen H., Wang S., Ma J., *et al.* High-Performance photodetectors based on lead-free 2D Ruddlesden–Popper perovskite/$MoS_2$ heterostructures. *ACS Appl. Mater. Interfaces* **11**, 8419-8427 (2019).

62. Yin L., Wang F., Cheng R., Wang Z., Chu J., Wen Y., *et al.* Van der Waals heterostructure devices with dynamically controlled conduction polarity and multifunctionality. *Adv. Funct. Mater.* **29**, 1804897 (2019).

63. Lv Q., Yan F., Wei X., Wang K. High-Performance, self-driven photodetector based on graphene sandwiched GaSe/$WS_2$ heterojunction. *Adv. Opt. Mater.* **6**, 1700490 (2018).

64. Wang H., Li Z., Li D., Xu X., Chen P., Pi L., *et al.* Junction field-effect transistors based on $PdSe_2$/$MoS_2$ heterostructures for photodetectors showing high responsivity and detectivity. *Adv. Funct. Mater.* **31**, 2106105 (2021).

65. Zhou X., Hu X., Zhou S., Song H., Zhang Q., Pi L., *et al.* Tunneling diode based on $WSe_2$/$SnS_2$ heterostructure incorporating high detectivity and responsivity. *Adv. Mater.* **30**, 1703286 (2018).

66. Cai Z., Cao M., Jin Z., Yi K., Chen X., Wei D. Large photoelectric-gating effect of two-dimensional van-der-Waals organic/tungsten diselenide heterointerface. *npj 2D Mater Appl* **2**, 21 (2018).

67. Jin B., Zuo N., Hu Z.-Y., Cui W., Wang R., Van Tendeloo G., *et al.* Excellent excitonic photovoltaic effect in 2D $CsPbBr_3$/CdS heterostructures. *Adv. Funct. Mater.* **30**, 2006166 (2020).

68. Zhang H., Zhang X., Liu C., Lee S.-T., Jie J. High-Responsivity, high-detectivity, ultrafast topological insulator $Bi_2Se_3$/Silicon heterostructure broadband photodetectors. *ACS Nano* **10**, 5113-5122 (2016).

69. Moun M., Singh R. Exploring conduction mechanism and photoresponse in P-GaN/n-$MoS_2$ heterojunction diode. *J. Appl. Phys.* **127**, 135702 (2020).

70. Wang Y., Zhang Y., Lu Y., Xu W., Mu H., Chen C., *et al.* Hybrid graphene–perovskite phototransistors with ultrahigh responsivity and gain. *Adv. Opt. Mater.* **3**, 1389-1396 (2015).

71. Ma C., Shi Y., Hu W., Chiu M.-H., Liu Z., Bera A., *et al.* Heterostructured $WS_2$/$CH_3NH_3PbI_3$ photoconductors with suppressed dark current and enhanced photodetectivity. *Adv. Mater.* **28**, 3683-3689 (2016).

72. Wu D., Wang Y., Zeng L., Jia C., Wu E., Xu T., *et al.* Design of 2D layered $PtSe_2$ heterojunction for the high-performance, room-temperature, broadband, infrared photodetector. *ACS Photonics* **5**, 3820-3827 (2018).

73. Yu Y., Zhang Y., Song X., Zhang H., Cao M., Che Y., *et al.* PbS-Decorated $WS_2$ phototransistors with fast response. *ACS Photonics* **4**, 950-956 (2017).

74. Kufer D., Nikitskiy I., Lasanta T., Navickaite G., Koppens F. H. L., Konstantatos G. Hybrid 2D–0D $MoS_2$–PbS quantum dot photodetectors. *Adv. Mater.* **27**, 176-180 (2015).

75. Hu C., Dong D., Yang X., Qiao K., Yang D., Deng H., *et al.* Synergistic effect of hybrid PbS quantum dots/2D-$WSe_2$ toward high performance and broadband phototransistors. *Adv. Funct. Mater.* **27**, 1603605 (2017).

76. Chen C., Qiao H., Lin S., Man Luk C., Liu Y., Xu Z., *et al.* Highly responsive $MoS_2$ photodetectors enhanced by graphene quantum dots. *Sci. Rep.* **5**, 11830 (2015).

77. Sun M., Fang Q., Xie D., Sun Y., Qian L., Xu J., *et al.* Heterostructured graphene quantum dot/$WSe_2$/Si photodetector with suppressed dark current and improved detectivity. *Nano Res.* **11**, 3233-3243 (2018).

78. Tong L., Huang X., Wang P., Ye L., Peng M., An L., *et al.* Stable mid-infrared polarization imaging based on quasi-2D tellurium at room temperature. *Nat. Commun.* **11**, 2308 (2020).

79. Huo N., Konstantatos G. Ultrasensitive all-2D $MoS_2$ phototransistors enabled by an out-of-plane $MoS_2$ pn homojunction. *Nat. Commun.* **8**, 572 (2017).

80. Lopez-Sanchez O., Lembke D., Kayci M., Radenovic A., Kis A. Ultrasensitive photodetectors based on monolayer $MoS_2$. *Nat. Nanotechnol.* **8**, 497-501 (2013).

81. Saran R., Curry R. J. Lead sulphide nanocrystal photodetector technologies. *Nat. Photon.* **10**, 81-92 (2016).

82. Berencén Y., Prucnal S., Liu F., Skorupa I., Hübner R., Rebohle L., *et al.* Room-temperature short-wavelength infrared Si photodetector. *Sci. Rep.* **7**, 43688 (2017).

83. Rogalski A. Infrared detectors: status and trends. *Prog. Quant. Electron.* **27**, 59-210 (2003).

84. Zhao Q., Gao F., Chen H., Gao W., Xia M., Pan Y., *et al.* High performance polarization-sensitive self-powered imaging photodetectors based on a p-Te/n-$MoSe_2$ van der Waals heterojunction with strong interlayer transition. *Mater. Horiz.* **8**, 3113-3123 (2021).

85. Tao L., Li S., Yao B., Xia M., Gao W., Yang Y., *et al.* Raman anisotropy and polarization-sensitive photodetection in 2D $Bi_2O_2Se$–$WSe_2$ heterostructure. *ACS Omega* **6**, 34763-34770 (2021).

86. Qiu Q., Huang Z. Photodetectors of 2D materials from ultraviolet to terahertz waves. *Adv. Mater.* **33**, 2008126 (2021).

87. Ling X., Huang S., Hasdeo E. H., Liang L., Parkin W. M., Tatsumi Y., *et al.* Anisotropic electron-photon and electron-phonon interactions in black phosphorus. *Nano Lett.* **16**, 2260-2267 (2016).

88. Kecik D., Bacaksiz C., Senger R. T., Durgun E. Layer- and strain-dependent optoelectronic properties of hexagonal AlN. *Phys. Rev. B.* **92**, 165408 (2015).
